# Preparation of alginate hydrogel microparticles by gelation introducing cross-linkers using droplet-based microfluidics: a review of methods

**DOI:** 10.1186/s40824-021-00243-5

**Published:** 2021-11-24

**Authors:** Cheng Zhang, Romain Grossier, Nadine Candoni, Stéphane Veesler

**Affiliations:** CNRS, Aix-Marseille Université, CINaM (Centre Interdisciplinaire de Nanosciences de Marseille), Campus de Luminy, Case 913, F-13288 Marseille Cedex 09, France

**Keywords:** Alginate, Hydrogel, Microparticle, Crosslinking, Droplet-based microfluidics

## Abstract

This review examines the preparation of alginate hydrogel microparticles by using droplet-based microfluidics, a technique widely employed for its ease of use and excellent control of physicochemical properties, with narrow size distribution. The gelation of alginate is realized “on-chip” and/or “off-chip”, depending on where cross-linkers are introduced. Various strategies are described and compared. Microparticle properties such as size, shape, concentration, stability and mechanical properties are discussed. Finally, we consider future perspectives for the preparation of hydrogel microparticles and their potential applications.

## Introduction

Hydrogel microparticles are widely used today, especially in biological and pharmaceutical applications. They are usually used as a matrix to encapsulate bioactive agents such as drugs, proteins, cells, etc. [2, 12, 30] in applications like drug delivery [1], cell culture and tissue engineering [49]. In addition, fluorescence-encoded hydrogel microparticles are extensively employed in multiplex bioassays [42, 62, 64]. Another important use is as cell-mimicking microparticles with similar size, shape, deformability and mechanical properties [18, 32, 59]. Hydrogels can be made of various biopolymers such as gelatine, agarose, alginate, pectin, etc. Alginate stands out because of its low cost, non-toxicity and ease of crosslinking [25].

With the increasing interest in alginate hydrogel microparticles, various preparation methods have been reported in the literature [27], including conventional emulsification [8], spray-drying [40], extrusion dripping [4, 24], microfluidics [39, 56, 60] and soft lithography [36]. The huge diversity of techniques and strategies can make it confusing to choose the right method. The present review focuses on a microparticle-producing technique widely used for its efficacy in controlling physicochemical properties: droplet-based microfluidics. After basic recalls on alginate chemistry and droplet-based microfluidics, the various strategies applied within this technique and the properties of the microparticles obtained are described in this review. We expect this paper useful for researchers who want to know what is possible to do with droplet-based microfluidics for the preparation of alginate hydrogel microparticles by introducing cross-linkers.

## Alginate hydrogel microparticles

Hydrogels are described as hydrophilic polymeric networks which can absorb and retain large amounts of water within the structure. The hydrogel network is formed by polymer crosslinking. When crosslinking is realized by molecular entanglement, ionic, H-bonding or hydrophobic forces, hydrogels are called physical or reversible gels. Otherwise, when covalent forces intervene, they are called chemical or permanent gels [6, 19].

### Alginate

Alginate is a natural polysaccharide. Although it can also be synthesized by several bacteria, all the commercially available alginate is produced from the extraction of brown algae [11]. Alginate is widely used in the biomedical field because it is biocompatible and non-toxic [25].

**Fig. 1:**
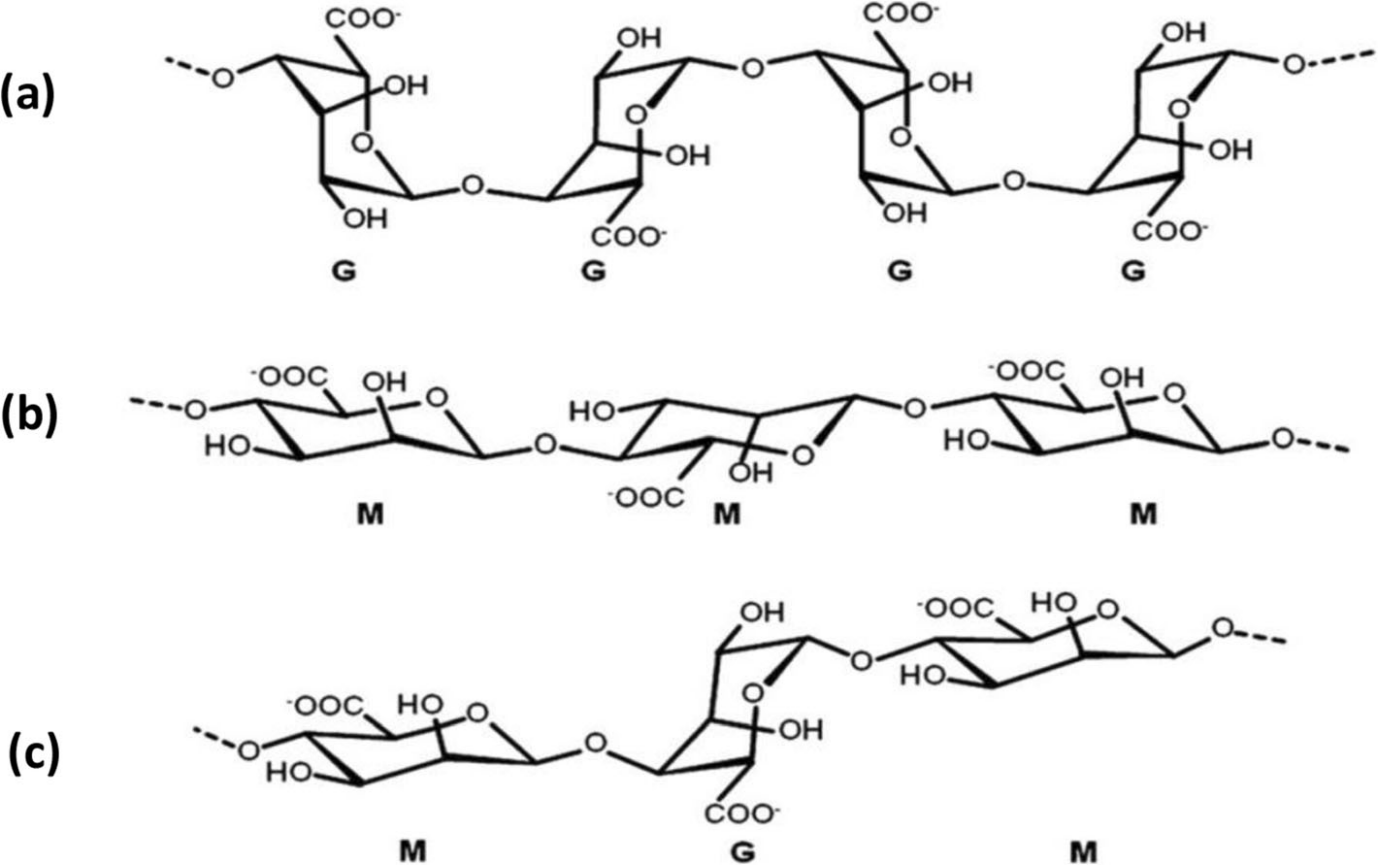
Chemical structures of **a** G-block, **b** M-block and **c** alternating G and M-blocks in alginate. Figure reprinted with permission from Reference [25]

Sodium alginate (Na-alginate) is the most widely used alginate salt. It dissolves in water to a viscous solution. Alginate is a linear copolymer containing β-D-mannuronate (M) and α-L-guluronate (G) residues (Fig. 1).

### Gelation of alginate

Alginate hydrogel is produced by gelation which is caused by covalent [14] or ionic crosslinking [15, 51]. Ionic crosslinking is more commonly used because of its simplicity and mild conditions. It can be carried out at room temperature or up to 100 °C, usually with divalent cations as cross-linkers. Calcium chloride is the most widely used [25], due to its non-toxicity [1] and availability.

**Fig. 2:**
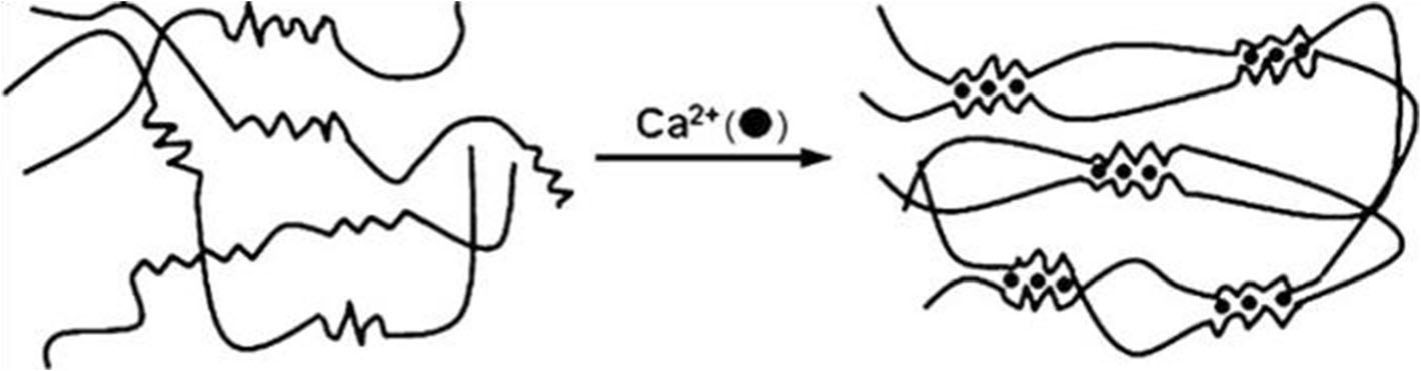
Schematic illustration of the “egg-box” model describing the ionic crosslinking of alginate by calcium cations. Figure reprinted with permission from Reference [26]

Only G-blocks (Fig. 1a) made of consecutive G residues can participate in ionic crosslinking because of their favorable spatial structure [15, 25]. Ionic crosslinking of alginate is described by the “egg-box” model [16] (Fig. 2).

In this review, we present various two-step methods of producing alginate hydrogel microparticles. First, sodium alginate droplets are generated using droplet-based microfluidics. Second, internal or external gelation transforms droplets into alginate hydrogel microparticles via different strategies. It should be noted that only ionic crosslinking is discussed herein. In internal gelation, the cross-linkers are inside the alginate droplet whereas in external gelation, the cross-linkers come from outside the alginate droplet.

## Droplet-based microfluidics

### Principle of droplet generation

Microfluidics is a technique used to manipulate fluids in channels of micrometric dimensions. Fluids are mixed by adding junctions that connect the channels. When immiscible or partially miscible fluids are mixed in the junction, microdroplets can be generated: this is called droplet-based microfluidics.

The principle is similar to that of conventional emulsification, which consists of blending two immiscible liquids. The advantage of droplet-based microfluidics is monodispersity and repeatability of droplets due to precise control over experimental conditions such as channel geometry, flow rates and viscosities of fluids, etc. [41, 46]. Furthermore, monodisperse droplets can be generated without using surfactant [28, 48, 59], which is impossible with conventional emulsification.

The droplets generated in droplet-based microfluidics can serve as microreactors to carry out physical, chemical or biological reactions [65]. Being small (nL to μL volume), they require a small quantity of reactants. As droplet composition can be made identical, numerous identical experiments can be performed, enabling a reliable statistical approach to data.

### Flow properties

In droplet-based microfluidics, a continuous fluid and a dispersed fluid are injected separately and then mixed in a junction. Fluids are Newtonian and droplets of the dispersed fluid (D) are generated in the flow of the continuous fluid (C). The physicochemical properties influencing droplet formation are density, dynamic viscosity, surface tension between the continuous and the dispersed fluids, velocity of the flows and characteristic dimensions of the microfluidic system, such as the diameter of channels (w) for cylindrical microfluidic systems. Based on these properties, fluid dynamics is characterized as follows:

1. Inertial forces and viscous forces are compared through the Reynolds number, calculated using the continuous fluid properties: density (ρ_C_), dynamic viscosity (μ_C_) and flow velocity (*v*_*C*_).

1 $$ \mathit{\operatorname{Re}}=\frac{\rho_C\times {v}_C\times \mathrm{w}}{\mu_C} $$

Typically for microfluidics, values of Re are lower than 1: the flow is laminar and the effect of inertia can be ignored. Thus, the average velocity v of a flow is evaluated from its volumetric flow rate Q and w as follows:

2 $$ v=\frac{Q}{\pi {\left(\mathrm{w}/2\right)}^2} $$

2- The generation of droplets in a microfluidic junction creates a free interface between the two fluids, characterized by the interfacial energy γ_CD_. The corresponding capillary effects are in competition with gravity effects. The length above which gravity effects dominate capillary effects is the capillary length l_c_:

3 $$ {l}_c=\sqrt{\frac{\gamma_{\mathrm{CD}}}{\Delta \uprho \times g}} $$

with g the gravity acceleration and Δρ the difference in density between the two fluids. For instance, with fluorinated oil FC70 as the continuous fluid and ethanol as the dispersed fluid, l_c_ is equal to 2.4 mm [63]. Hence gravity does not influence the deformation of the interfaces in millimetric or sub-millimetric channels.

3- Shear stress and interfacial energy are compared through the capillary number Ca. When generating droplets of a dispersed fluid in a continuous fluid, Ca is usually calculated using *v*_*C*_ and μ_C_ of the continuous fluid, and γ_CD_ of the interface between the continuous and the dispersed fluid:

4 $$ \mathrm{Ca}=\frac{\mu_C\times {v}_C}{\gamma_{\mathrm{CD}}} $$

### Microfluidic geometry

Microfluidic devices can be in the form of either chips with microchannels and junctions produced by soft lithography, or an assembly of capillaries and junctions [37]. In terms of materials, polydimethylsiloxane (PDMS) is the most commonly used for microfluidic chips [28, 60]. For capillaries, both glass [5, 20] and fluoropolymer can be used [48, 59].

**Fig. 3:**
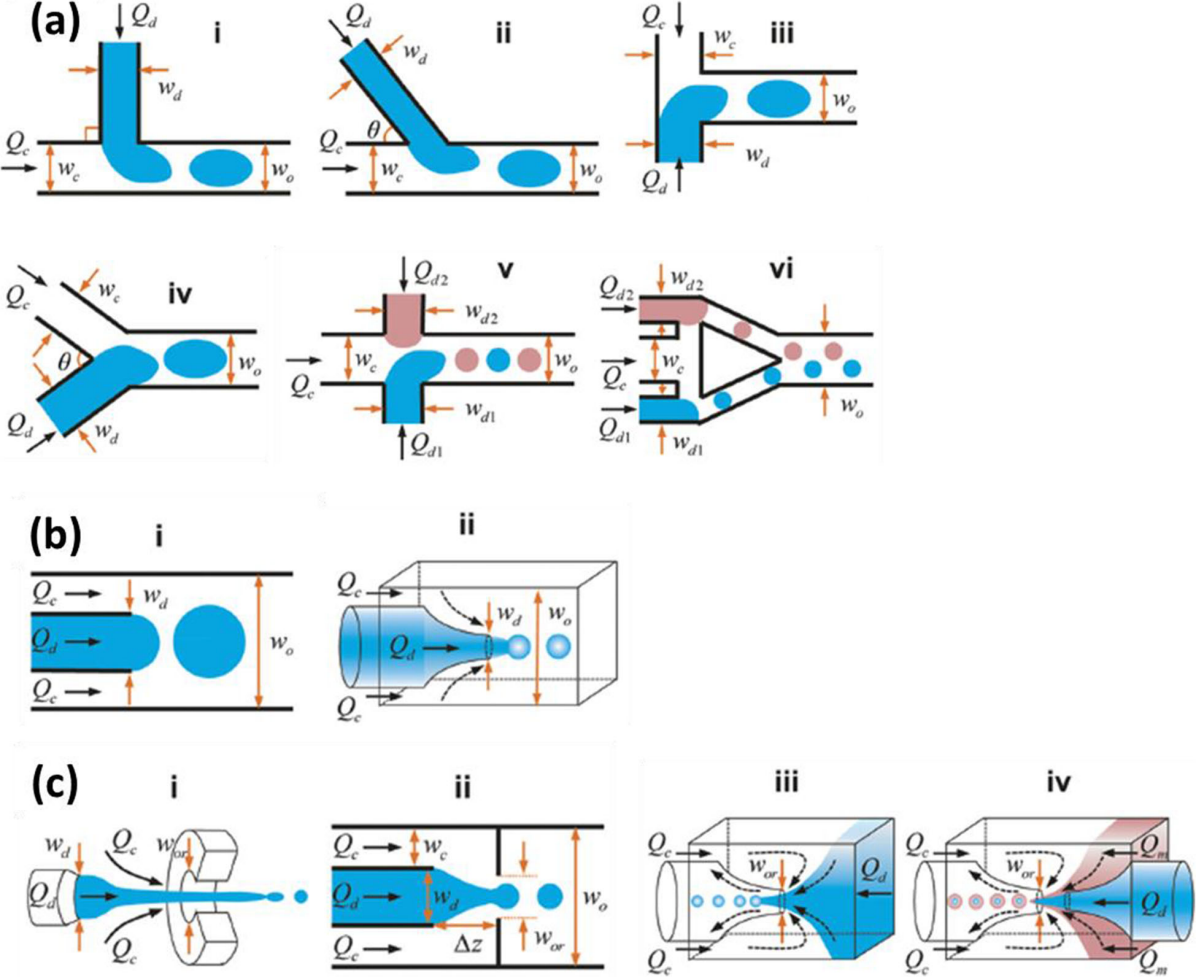
Schematic illustrations **a** “cross-flowing”, **b** “co-flow” and **c** “flow-focusing” geometries for a microfluidic device. ***Q*** and ***w*** denote respectively flow rate and channel width. Subscripts ***d***, ***c***, ***o*** and ***or*** denote respectively dispersed fluid, continuous fluid, outlet channel and orifice. Figure reprinted with permission from Reference [65]

The channel geometry of a microfluidic device influences droplet generation. Three frequently used geometries are “cross-flow”, “co-flow” and “flow-focusing” (Fig. 3).

#### Cross-flowing

For cross-flowing geometry, continuous fluid and dispersed fluid mix with an angle ***θ*** (0° < ***θ*** ≤ 180°) at the junction (Fig. 3a). Where the two fluids meet, first an interface is formed due to the immiscibility of the two fluids. Shear force then pushes the head of the dispersed fluid into the continuous fluid until a part breaks off: the droplet is formed. Then it circulates in the channel of the continuous fluid [46].

Cross-flowing geometry is often called T-junction geometry, where two fluids flow orthogonally (Fig. 3a i). However, other shapes of junctions can also be used, such as a junction with an arbitrary angle ***θ*** (Fig. 3a ii), or a Y-shaped junction (Fig. 3a iv). For two fluids facing each other (***θ*** = 180°, Fig. 3a iii), the geometry is called “head-on”. A combination of two junctions (Fig. 3a v, vi) can also be used to introduce two different dispersed fluids and one continuous fluid. Cross-flowing geometry is widely used due to its ease of assembly and handling [41, 65].

#### Flow-focusing

For co-flow geometry, two immiscible fluids flow in two concentric channels (Fig. 3b). Droplets are formed at the outlet of the inner channel. Flow-focusing geometry is actually similar to co-flow geometry. The distinction presented in the literature is somewhat ambiguous [65], leading some to consider flow-focusing as a special co-flow geometry [41]. For flow-focusing geometry (Fig. 3c), two immiscible fluids are focused through an orifice, which allows smaller droplets to be generated than with co-flow geometry.

### Droplet generation regime

**Fig. 4:**
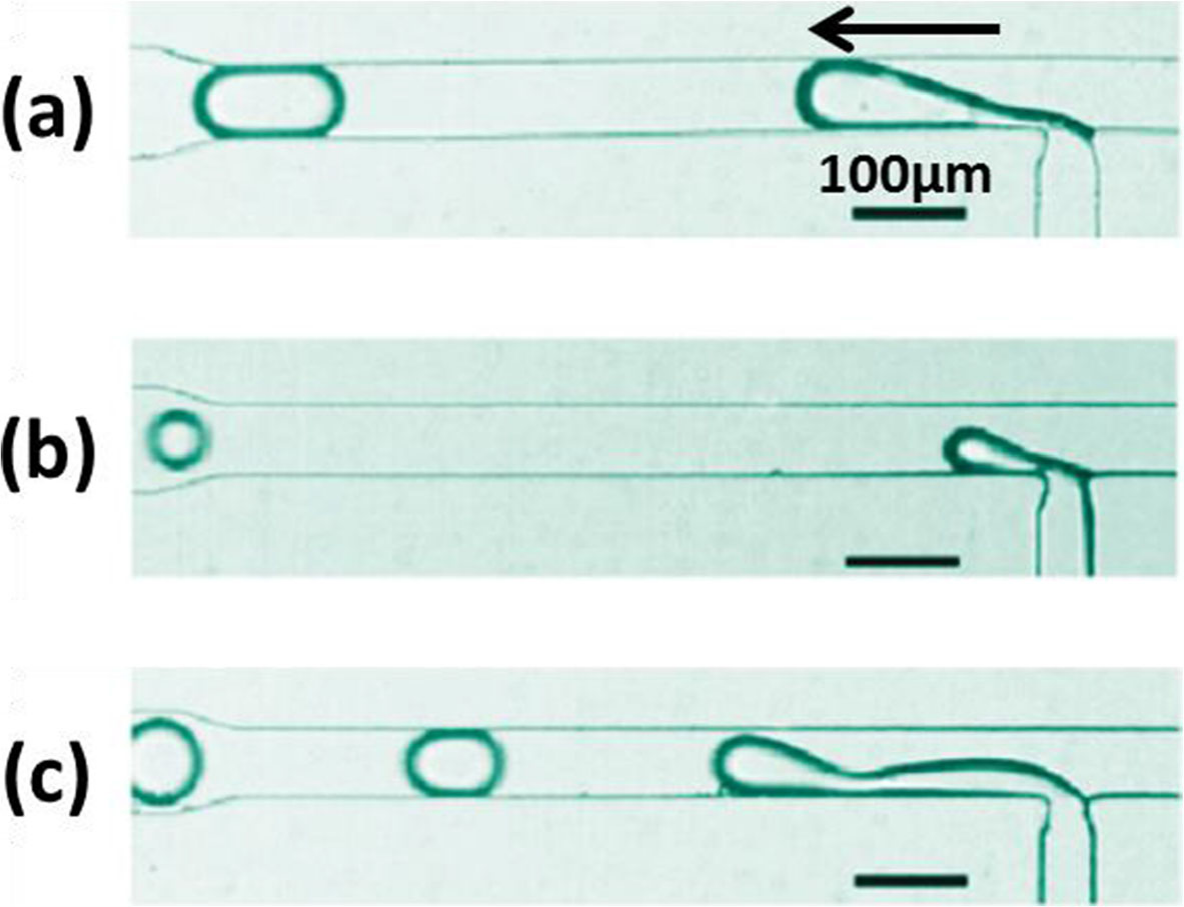
Three break-off mechanisms of droplet generation with a cross-flowing geometry: **a** squeezing, **b** dripping and **c** jetting. The arrow indicates the droplet flow direction. Figure reprinted with permission from Reference [57]. Copyright 2010 American Chemical Society

For each geometry, droplets can be generated following three different break-off mechanisms. The transition from one mechanism to another can be achieved by varying capillary numbers Ca [65]. Figure 4 shows an example of three mechanisms for a cross-flowing geometry.

#### Squeezing

As Fig. 4a shows, as it is injected into the principal channel, the dispersed fluid is pushed forward by the continuous fluid. A thin “neck” is thus formed. Because the continuous fluid applies weak shear force, the forming droplet reaches the opposing channel wall without breaking off. The neck becomes thinner until it breaks, so that a plug-shaped droplet confined by channel wall is formed. Squeezing mechanism appears when Ca is low (Ca ≤ 0.01) [10].

#### Dripping

As Fig. 4b shows, the shear force applied is now higher. The forming droplet breaks off before touching the opposing channel wall. A spherical droplet is formed with a diameter smaller than that of the channel. This dripping mechanism appears at a higher Ca (Ca ≥ 0.02) [10].

#### Jetting

As Fig. 4c shows, a liquid jet is emitted from the dispersed fluid channel. It flows and remains attached to the channel wall, due to a strong shear force from the continuous fluid [9]. The jet breaks up into droplets at the end because of Rayleigh-Plateau instability [65]. Droplets of polydisperse sizes are formed. This jetting mechanism appears at the highest Ca (Ca ≈ 0.2).

## Gelation

### Internal gelation

For internal gelation, cross-linkers come from inside the alginate droplets and are either soluble or insoluble/slightly soluble in water. In this approach, cross-linkers are always introduced in the microfluidic device.

#### Water-soluble cross-linkers

With water-soluble cross-linkers such as barium chloride (BaCl_2_) and calcium chloride (CaCl_2_), alginate is crosslinked directly at the interior of droplets. These agents can be mixed with Na-alginate before or after droplet generation.

##### Mixing cross-linkers before droplet generation

A first category of strategies is based on mixing water-soluble cross-linkers with Na-alginate before droplet generation. The cross-linkers used in these studies are BaCl_2_ and CaCl_2_; these strategies are summarized in Table 1.

**Table 1:** Internal gelation with water-soluble cross-linkers: mixing cross-linkers with Na-alginate before droplet generation

References		Trivedi et al., 2009 [48]	Zhang et al., 2006 [60]	Rondeau and Cooper-White, 2008 [39]	Present review	Present review
**Droplet generation**	**Concentration of Na-alginate**	1 wt%	0.5 wt%	0.5 wt%	0.06 wt%	0.006 wt%
	**Continuous fluid**	Silicone oil	Mineral oil	DMC^a^	DMC^a^	DMC^a^
	**Use of surfactant**	NO	Span 80	Not mentioned	NO	NO
	**Geometry**	Flow-focusing	Flow-focusing	Flow-focusing	Cross-flowing	Cross-flowing
	**Microfluidic material**	Fluoropolymer tubing and junctions	PDMS chip	PDMS chip	Fluoropolymer tubing and junctions	Fluoropolymer tubing and junctions
**Internal gelation by mixing** **Na-alginate and water-soluble cross-linker**	**Cross-linker**	BaCl_2_ (50 mM)	CaCl_2_ (0.1 wt%)	CaCl_2_ (0.25 wt%)	CaCl_2_ (0.06 wt%)	CaCl_2_ (0.002 wt%)
	**Geometry**	Flow-focusing	Cross-flowing	Flow-focusing	–	Cross-flowing
	**Mixing**	Before droplet generation	During droplet generation	Before droplet generation	Off-line, before droplet generation	During droplet generation

^a^Continuous and dispersed fluids partially miscible - DMC (Dimethyl Carbonate)

Trivedi et al. worked on cell encapsulation by alginate hydrogel microparticles [48]. For the preparation of microparticles, an aqueous solution of cell-containing Na-alginate (1%) and a solution of BaCl_2_ (50 mM) were injected into the capillary and mixed via a Y-shaped junction. At the exit from the mixing region, highly viscous silicone oil (10 cSt) without surfactant was injected by flow-focusing in order to generate droplets. However, the mixing of Na-alginate and barium cations triggered ionic crosslinking, causing gelation in the mixing region which impacted droplet generation. Finally, instead of generating droplets as expected, a jet of gel was produced with a partially formed droplet head and a long gelatinous tail.

**Fig. 5:**
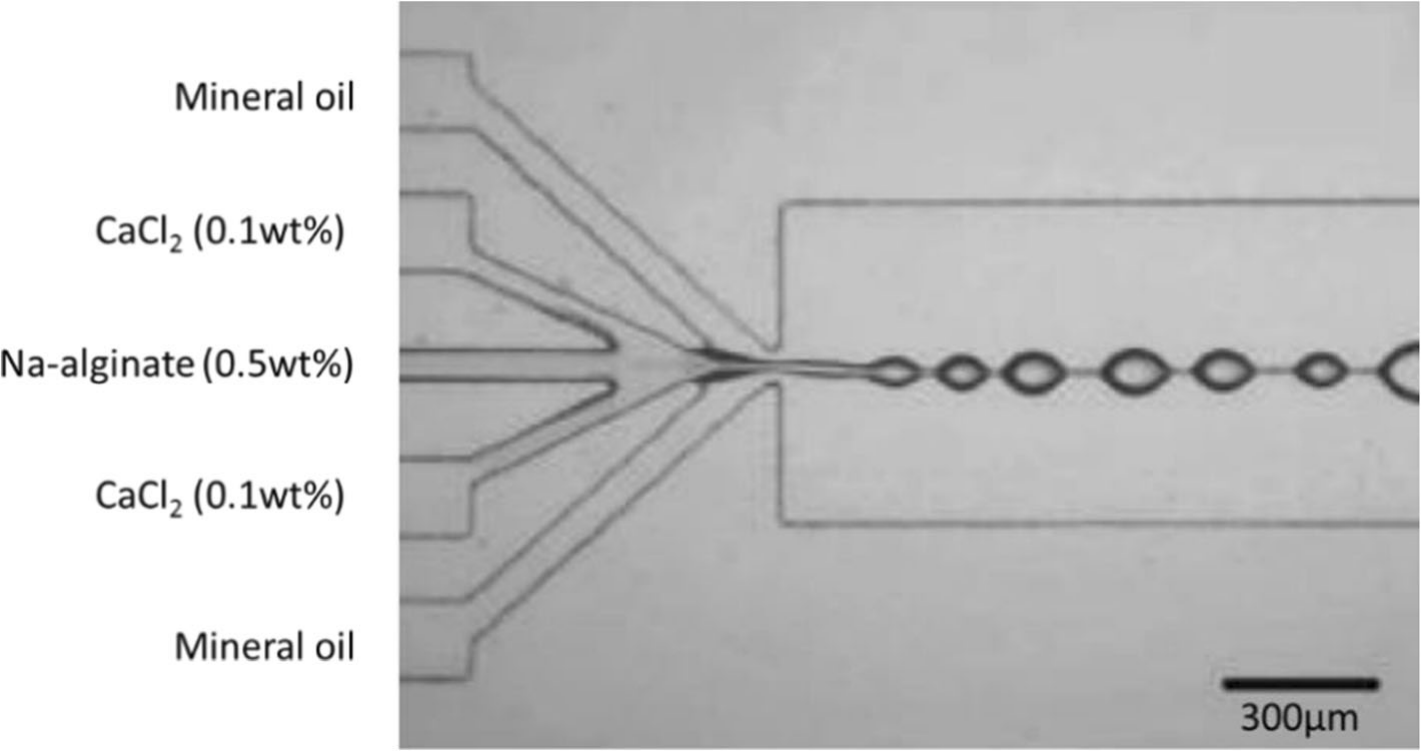
Image of connected knots formed after mixing Na-alginate and CaCl_2_ solutions in mineral oil with surfactant, in a PDMS-based microfluidic chip. Figure reprinted with permission from Reference [60]. Copyright 2006 American Chemical Society

To deal with this issue, the mixing region can be reduced before droplet generation, as Zhang et al. did [60] Using a 5-channel microfluidic device, they mixed Na-alginate fluid (0.5 wt%), CaCl_2_ fluids (0.1 wt%) and mineral oil fluids with a surfactant (Span 80, no concentration mentioned) as shown in Fig. 5. Droplets were generated by co-flow. However, instead of producing discrete droplets, a line of knots connected with each other was formed. This phenomenon persisted with a wide range of flow rates of oil due to viscosity which increased instantly when Na-alginate and CaCl_2_ were mixed, because of rapid gelation. It was therefore impossible to generate droplets at the junction, despite the use of surfactant.

**Fig. 6:**
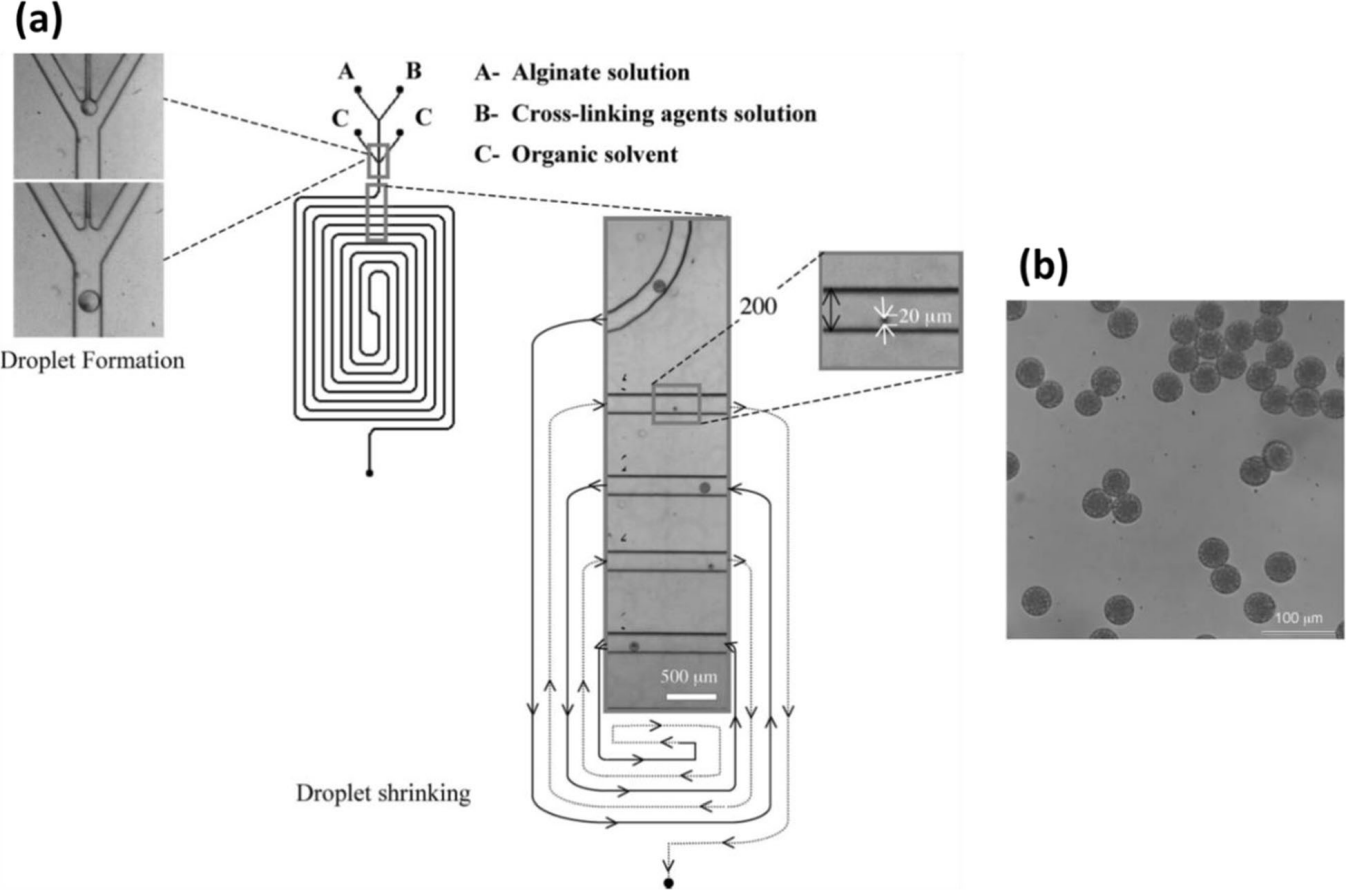
**a** Schematic diagram of a PDMS-based microfluidic device using DMC as the continuous fluid in which water is partially soluble. Droplet shrinkage is observed for an initial concentration of Na-alginate of 0.5 wt%. **b** Micrograph of Ca-alginate hydrogel microparticles collected in an aqueous solution of CaCl_2_. Figure reprinted with permission from Reference [39]. Copyright 2008 American Chemical Society

The problem can be solved by using low concentrations of Na-alginate and CaCl_2_ solutions. In this case, gelation proceeds after droplet generation and is enhanced by using partially miscible fluids. Rondeau and Cooper-White used Dimethyl carbonate (DMC) as the continuous fluid [39] (Fig. 6). The solubility of water in DMC is about 3 wt% at room temperature [43]. Aqueous solutions of Na-alginate (0.5 wt%) and CaCl_2_ (0.25 wt%) were injected respectively from inlets A and B (Fig. 6a). After a short pre-gelation channel, DMC was injected from inlet C. Na-alginate/CaCl_2_ droplets were generated in DMC (no mention of surfactant usage) by flow-focusing. Along the serpentine channel, because of the low solubility of water in DMC, water diffused gradually from droplets into DMC, causing the shrinkage of droplets along the channel. Internal gelation occurred at the same time. Microparticles with a diameter of 20 μm were observed at the outlet of the channel and collected in an aqueous solution of CaCl_2_ (2 N) to reinforce the gelation (Fig. 6b). The diameter of Ca-alginate hydrogel microparticles was influenced by the experimental parameters such as the initial concentration of Na-alginate, flow rates of fluids and channel size. To be precise, smaller Ca-alginate hydrogel microparticles can be obtained by using a less concentrated Na-alginate solution, a higher flow rate ratio between the continuous fluid and the dispersed fluid, or a narrower channel. However, DMC is also slightly soluble in water, with a solubility of 12.7 wt% at 20 °C [43]. Thus, during diffusion of water from droplets into DMC, DMC can also diffuse into droplets. This means that, after gelation, DMC can be captured inside alginate hydrogel microparticles. Additional work measuring the amount of DMC residue within microparticles could open the way to further applications.

**Fig. 7:**
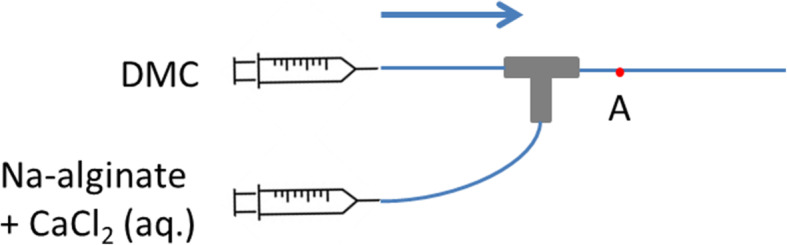
Schematic diagram of the generation of droplets of Ca-alginate with DMC as the continuous fluid and an aqueous mixed solution of Na-alginate and CaCl_2_ as the dispersed fluid. The device is composed of polyether ether ketone (PEEK) junctions and Teflon-like tubing (IDEX Health and Science). The arrow indicates the flow direction

Following the work of Rondeau and Cooper-White, we tested, in a T-junction (Fig. 7), the direct generation of droplets of Ca-alginate in DMC without surfactant from a mixture of more diluted Na-alginate and CaCl_2_ solutions (both at 0.06 wt% after mixing). However, this solution was not clear and local gelation was occasionally observed with the naked eye. When these gels entered the channel, droplets were generated in a discontinuous way. This indicates that, even at very low concentrations, thorough mixing of Na-alginate and CaCl_2_ solutions leads to gelation, disturbing droplet generation.

**Fig. 8:**
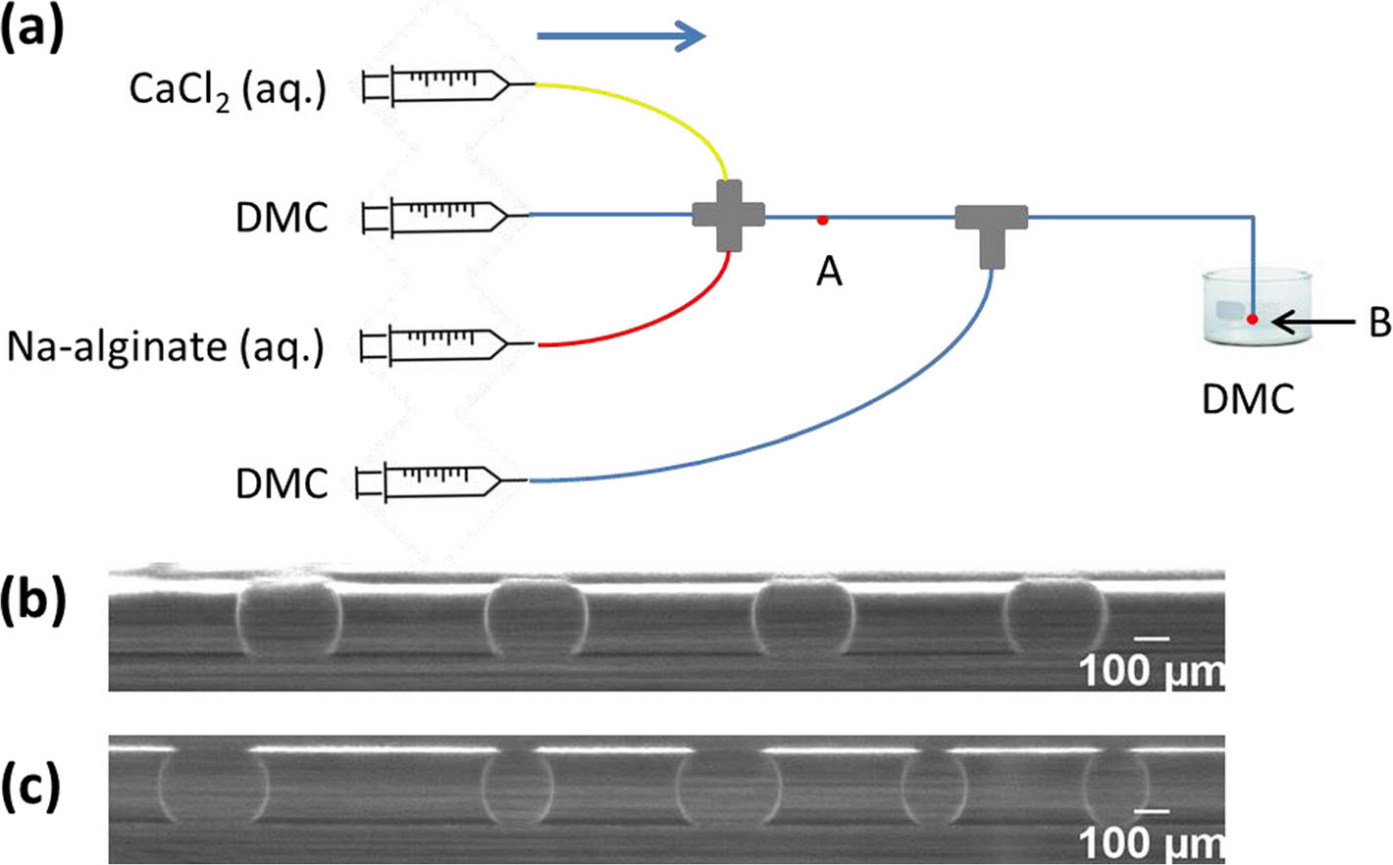
**a** Schematic diagram of the generation of Ca-alginate droplets in DMC using a cross-junction. The T-junction served to introduce DMC as a spacer to increase the distance between droplets. The device is composed of polyether ether ketone (PEEK) junctions and Teflon-like tubing (IDEX Health and Science). Micrograph of droplets observed at point A when **b** droplet generation was not disturbed by introducing the spacer and **c** when it was disturbed

In a microfluidic device (Fig. 8) of similar design to Zhang et al. [59], we were able to generate discrete droplets by using extremely diluted solutions of Na-alginate (0.006 wt%) and CaCl_2_ (0.002 wt%). The continuous fluid was DMC without surfactant. Droplets were observed after the cross-junction (point A in Fig. 8a). Since they were relatively close to each other in the channel, causing coalescence at the outlet (point B in Fig. 8a), a second flow of DMC was introduced as a spacer using a T-junction. When the second DMC flow rate was relatively low, the generation of droplets upstream was not disturbed, so that droplets were uniform (Fig. 8b). However, the coalescence at the outlet persisted. Thus, high second DMC flow rates were applied to sufficiently increase the distance between droplets. Nevertheless, this quickly disturbed the generation of droplets upstream, as indicated by heterogeneities in droplet size and frequency (Fig. 8c). Using surfactant would prevent droplet coalescence.

To summarize (Table 1), authors mixed highly concentrated solutions of Na-alginate and water-soluble cross-linkers before droplet generation to make them gelate. However, droplet generation was hindered by rapid gelation and microparticles were difficult to obtain. To delay gelation, less concentrated solutions of Na-alginate and water-soluble cross-linkers were mixed before droplet generation, in the microfluidic device or off-line. Then, concentrations were increased after droplet generation by diffusion of water from the droplets to the continuous fluid, due to their partial miscibility with water. Hence, gelation proceeded slowly with droplet shrinkage. However, mixing cross-linkers with Na-alginate before droplet generation led to heterogeneities in droplet size and frequency.

##### Mixing cross-linkers after droplet generation

To delay gelation, water-soluble cross-linkers need to be mixed with Na-alginate only after droplet generation. Studies doing so, and which also use BaCl_2_ and CaCl_2_ as cross-linkers, are summarized in Table 2.

**Table 2:** Internal gelation with water-soluble cross-linkers: mixing cross-linkers with Na-alginate after droplet generation

Reference		Xu et al., 2008 [54]	Liu et al., 2006 [28]	Trivedi et al., 2010 [47]
**Droplet generation**	**Concentration of Na-alginate**	2 wt%	2 wt%	1 wt%
	**Continuous fluid**	octyl alcohol oil	soybean oil	silicone oil
	**Use of surfactant**	Not mentioned	NO	NO
	**Geometry**	Flow-focusing	Flow-focusing	Flow-focusing
	**Microfluidic material**	PMMA chip	PDMS chip	Fluoropolymer tubing and junctions
**Internal gelation by mixing Na-alginate and water-soluble cross-linker**	**Cross-linkers**	CaCl_2_ (2 wt%)	CaCl_2_ (2 wt%)	BaCl_2_ (50 mM)
	**Geometry**	Cross-flowing	Expansion chamber	Cross-flowing
	**Mixing**	Coalescence of flows	Coalescence of droplets	Coalescence of droplets with flow

**Fig. 9:**
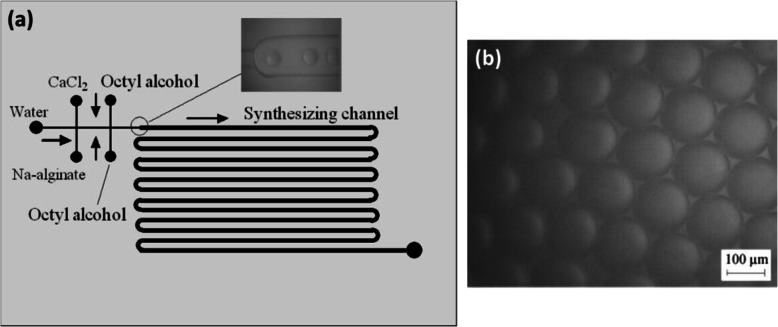
**a** Schematic diagram of Ca-alginate hydrogel microparticles prepared in a PMMA based microfluidic device. **b** Micrographs of Ca-alginate hydrogel microparticles. Figure reprinted with permission from Reference [54]

Xu et al. prevented rapid gelation by delaying the direct contact between Na-alginate and calcium cations [54]. In a first cross-junction, two face-to-face channels were used to introduce CaCl_2_ (2 wt%) and Na-alginate (2 wt%) solutions (Fig. 9a) perpendicularly to a flow of water. Thus, after the first cross-junction, a flow of water (acting as a buffer) separates the flows of Na-alginate and CaCl_2_. Then octyl alcohol oil (no mention of surfactant) was injected at a second cross-junction. Droplets of Na-alginate/CaCl_2_ were generated by flow-focusing. In the “synthesizing channel” (Fig. 9a), within each droplet, mixing Na-alginate and CaCl_2_ induced internal gelation. In this way droplets were transformed into Ca-alginate hydrogel microparticles (Fig. 9b). For this strategy, the size of Ca-alginate hydrogel microparticles is entirely dependent on the experimental conditions, such as flow rates of fluids and channel size. Manipulation of microparticles is difficult if their diameter is smaller than 10 μm.

**Fig. 10:**
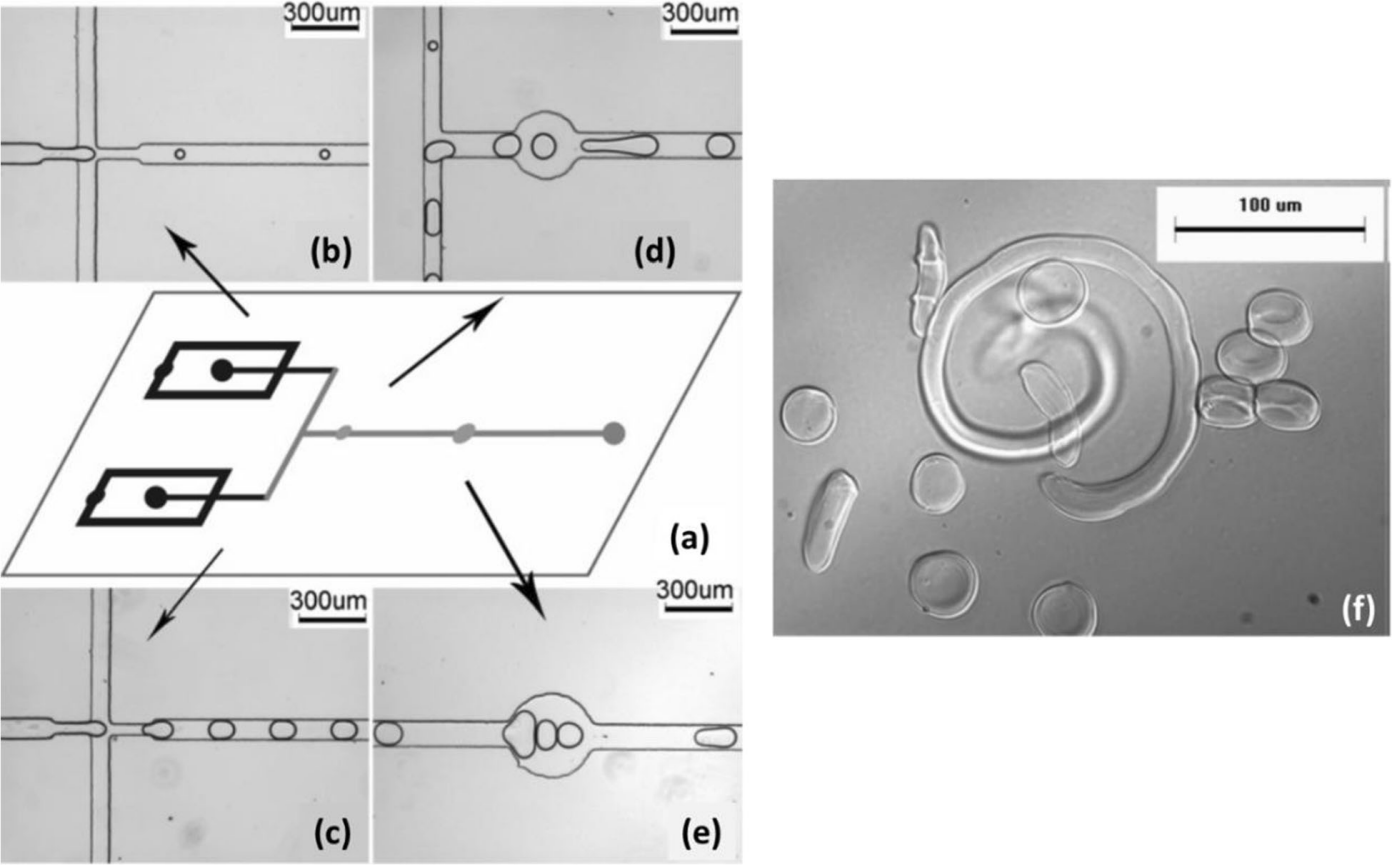
**a** Schematic diagram of the PDMS-based microfluidic device. **b** Flow-focusing channel to generate alginate droplets. **c** Flow-focusing channel to generate CaCl_2_ droplets. **d** T-junction followed by a first circular expansion chamber. **e** A second circular expansion chamber. **f** Ca-alginate hydrogel microparticles of different shapes and sizes. Figure reprinted with permission from Reference [28]. Copyright 2006 American Chemical Society

Another strategy to delay gelation was carried out by Liu et al. [28] involving coalescence of Na-alginate droplets with CaCl_2_ droplets generated separately. First, on a microfluidic chip (Fig. 10a), Na-alginate (2 wt%) droplets (Fig. 10b) and CaCl_2_ (2 wt%) droplets (Fig. 10c) were generated in soybean oil without surfactant by flow-focusing using two independent cross-junctions. Then droplets converged via a T-junction (Fig. 10d) followed by two successive circular expansion chambers (Fig. 10d, e). Thus, droplets could collide either at the T-junction or in circular chambers. Within the coalesced droplets, Na-alginate was crosslinked by calcium cations forming Ca-alginate hydrogel microparticles. With different flow rates and channel geometries, various shapes and sizes of microparticles could be produced (Fig. 10f). Nevertheless, the design of circular expansion chambers gives rise to local changes in flow velocity. Droplet circulation can be disturbed, thereby affecting homogeneity in droplet shape, size and frequency.

Droplets could also be coalesced by exploiting physicochemical parameters between the continuous fluid and the dispersed fluid. In the work of Trivedi et al., droplets of Na-alginate (1 wt%) containing cells were generated upstream in a highly viscous silicone oil (10 centistoke) by flow-focusing without surfactant [47, 48]. An aqueous solution of BaCl_2_ (50 mM) was injected downstream by a T-junction. With the help of dye, observations at the T-junction indicated that BaCl_2_ fluid merged spontaneously with Na-alginate/cells droplets, instead of forming independent BaCl_2_ droplets. However, this strategy lacks flexibility. The expected coalescence happens only when appropriate solvents are used. For instance, when using low-viscosity and low-interfacial energy γ_CD_ soybean oil, independent droplets of BaCl_2_ were observed. They coalesced downstream with Na-alginate/cells droplets. This implied that successful coalescence of droplets could only take place with appropriate interfacial energy and viscosity [48].

#### Water-insoluble or weakly soluble cross-linkers

To delay gelation, Na-alginate can be mixed with water-insoluble or weakly soluble cross-linkers, in water. This will not lead to instant gelation since there are no available cations in water. In the case of cross-linkers which are pH-sensitive, such as calcium carbonate (CaCO_3_) and calcium-ethylenediaminetetraacetic acid (Ca-EDTA) complex, an acid is used in the continuous fluid to release the cations from inert cross-linkers. Therefore, gelation by the available cations happens after droplet generation. These strategies are summarized in Table 3.

**Table 3:** Internal gelation with water-insoluble or weakly soluble cross-linkers which are pH sensitive

References		Zhang et al., 2007 [61]	Akbari and Pirbodaghi, 2014 [3]	Yu et al., 2019 [56]	Utech et al., 2015 [49]
**Droplet generation**	**Concentration of Na-alginate**	2 wt%	1,5 wt%	2 w/v%	2 wt%
	**Continuous fluid**	Soybean oil	Fluorocarbon oil	Mineral oil	Fluorinated carbon oil
	**Use of surfactant**	Span 80	Fluorinated surfactant	Span 80	Biocompatible surfactant
	**Geometry**	Flow-focusing	Flow-focusing	Flow-focusing	Flow-focusing
	**Microfluidic material**	PDMS chip	PDMS chip	PDMS chip	PDMS chip
**Internal gelation by mixing Na-alginate and water-insoluble or weakly soluble cross-linkers**	**Cross-linkers**	CaCO_3_ (0,1 wt%)	CaCO_3_ (35 mM)	CaCO_3_ (200 mM)	Ca-EDTA (50 mM)
	**Mixing**	Off-line, before droplet generation	Before droplet generation	Off-line, before droplet generation	Off-line, before droplet generation
	**Gelation by acid addition**	In continuous fluid	In continuous fluid	In collecting fluid	In continuous fluid

**Fig. 11:**
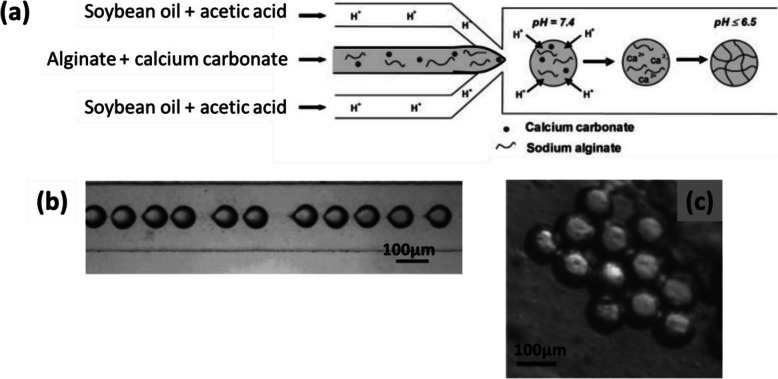
**a** Schematic diagram of the preparation of Ca-alginate hydrogel microparticles by using CaCO_3_ to perform internal gelation of alginate in a PDMS-based microfluidic device. Micrograph of **b** droplets generated in the channel and **c** Ca-alginate hydrogel microparticles collected in oil. Figure reprinted with permission from Reference [61]

In the work of Zhang et al. [61], fine particles of CaCO_3_ (0.1 wt%) were dispersed in an aqueous solution of Na-alginate (2 wt%). Soybean oil with a surfactant (Span 80, 3 wt%) and containing acetic acid (5 wt%) was used as the continuous fluid (Fig. 11a). Droplets of Na-alginate/CaCO_3_ were generated by flow-focusing in soybean oil/acetic acid (Fig. 11b). Droplets pH decreased because of the acetic acid in the oil. As a result, calcium cations were released from CaCO_3_, causing internal gelation of the alginate. Finally, Ca-alginate hydrogel microparticles were collected in oil (Fig. 11c). However, when collected on a substrate, they had a “pancake” shape and were soluble in aqueous solution owing to insufficient gelation. No improvement was observed from increasing the concentration of acetic acid or that of CaCO_3_. Moreover, a higher concentration of CaCO_3_ particles would give rise to their aggregation in the channel [61]. The mechanical properties of the microparticles could not therefore be improved.

**Fig. 12:**
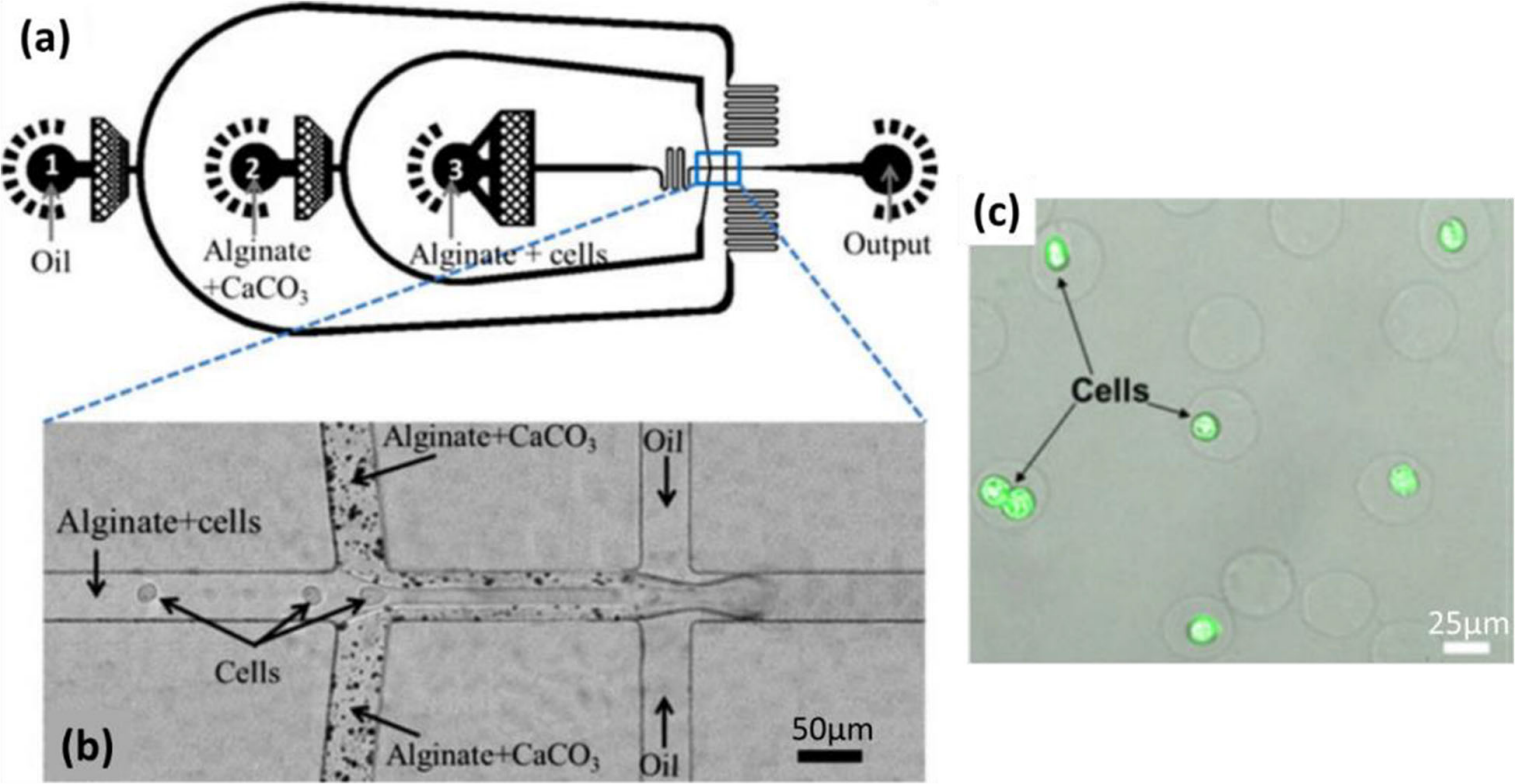
**a** Schematic diagram of a PDMS-based microfluidic device for the generation of droplets. **b** Micrograph of the two cross-junctions in the microfluidic device. **c** Confocal microscopic image of Ca-alginate hydrogel microparticles, some with cells encapsulated (Green fluorescence represents live cells stained by calcein AM). Figure reprinted with permission from Reference [3]

The same principle was also applied by Akbari and Pirbodaghi to prepare cell-encapsulating microparticles (Fig. 12) [3]. At a first T-junction, a fluid of Na-alginate (1.5 wt%) containing cells flowed into the middle channel (Fig. 12a), while the Na-alginate fluid (1.5 wt%) containing CaCO_3_ nanoparticles (35 mM) was introduced by two side channels (Fig. 12b). This geometry was used to create a coaxial stream while avoiding direct mechanical contact between cells and the potentially damaging CaCO_3_ particles. At a second T-junction, fluorocarbon oil with surfactant (fluorinated surfactant, 1 wt%) was injected. Droplets of Na-alginate/cells/CaCO_3_ were then generated by flow-focusing. After droplet collection, acetic acid (0.1 vol%) dissolved in oil was added to release calcium cations within droplets, causing gelation of alginate. Droplets were thus transformed into Ca-alginate hydrogel microparticles, some with cells encapsulated (Fig. 12c). However, the mixture of CaCO_3_ and Na-alginate was not homogeneous, which can be seen from Fig. 12b. Thus, the varying amounts of CaCO_3_ influenced the degree of gelation in each droplet, yielding Ca-alginate hydrogel microparticles with different mechanical properties. This issue is not discussed by Akbari and Pirbodaghi [3]. Furthermore, not all microparticles encapsulated cells, for reasons not explored in the article. Sorting is therefore required after the preparation of microparticles, which complicates the procedure.

**Fig. 13:**
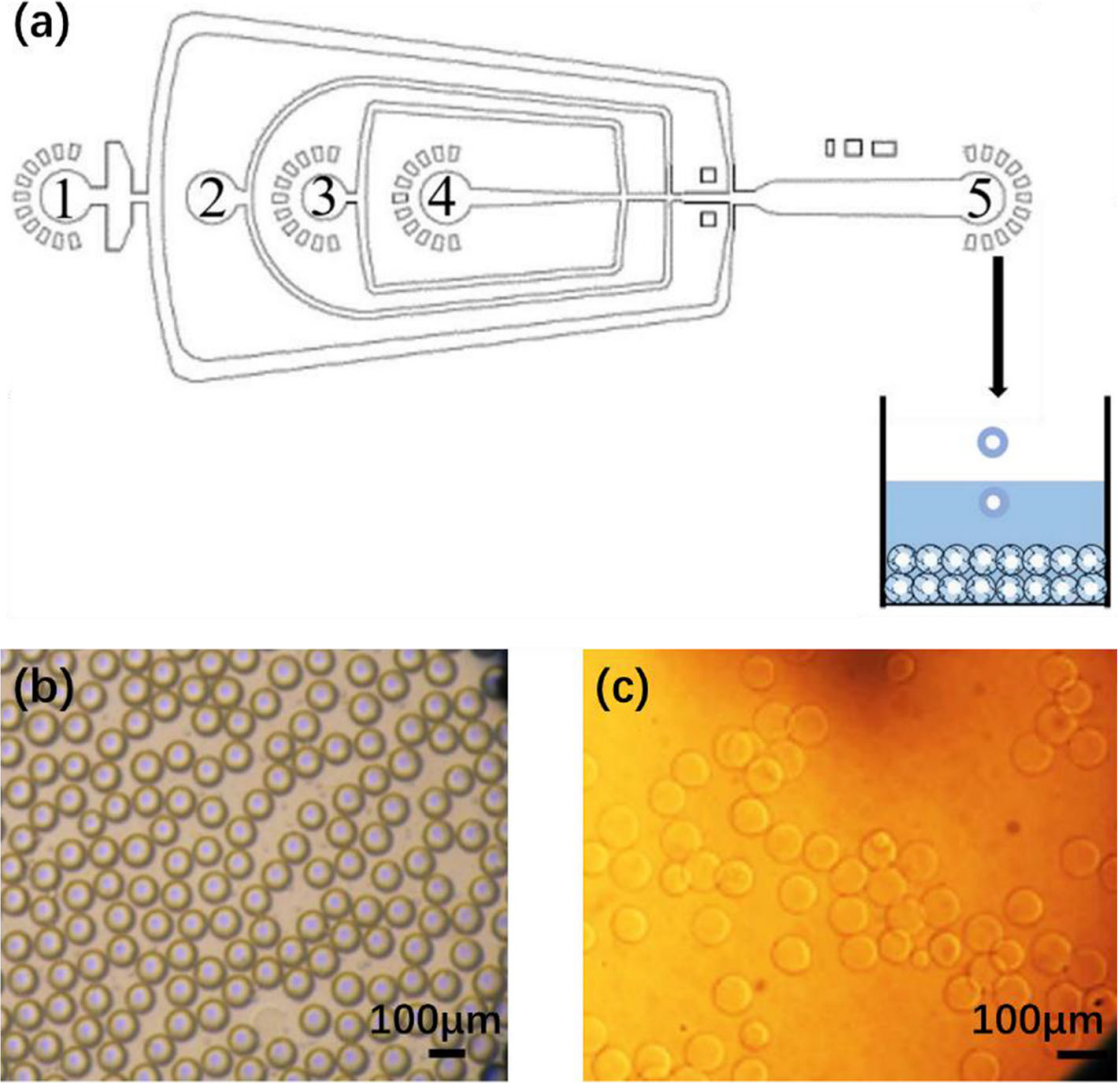
**a** Schematic diagram of a PDMS-based microfluidic device for the preparation of antigen-core alginate-shell microparticles. Inlet 1: Mineral oil with 3 wt% Span 80 and 0.2 vol% acetic acid; Inlet 2: Mineral oil with 3 wt% Span 80; Inlet 3: 2 w/v% alginate solution containing 200 mM CaCO_3_; Inlet 4: an antigen or protein aqueous solution. Micrographs of Ca-alginate hydrogel microparticles in **b** oil and **c** water. Figure reprinted with permission from Reference [56]

Combining the strategy of Zhang et al. and Akbari and Pirbodaghi to conduct gelation both in the microfluidic device and in the collection bath, Yu et al. [56] produced Ca-alginate hydrogel microparticles for protein encapsulation. First, from inlet 4 (Fig. 13a), an aqueous solution of antigen or protein was injected. It co-flowed with another aqueous solution of alginate (2 w/v%) mixed with CaCO_3_ particles (200 mM) and injected from inlet 3. Mineral oil with Span 80 added was injected from inlet 2 as a continuous fluid. In the flow-focusing channel, droplets containing alginate, CaCO_3_ and protein were formed. From inlet 1, another continuous fluid, mineral oil containing Span 80 and acetate acid, was introduced. When the acetate acid diffused into droplets, calcium cations were released. The alginate was then crosslinked, leading to preliminary gelation. The droplets were collected in an aqueous solution of CaCl_2_ (0.27 M) to enhance gelation. In the end, spherical hydrogel microparticles were formed, with protein encapsulated (Fig. 13b-c). According to the authors, the preliminary gelation in the microchannel prevented the deformation that occurs when droplets are collected directly in an aqueous solution of CaCl_2_. As mentioned above, however, since CaCO_3_ is not soluble in water, a high concentration of CaCO_3_ will clog the microchannel. Thus, the scope for preliminary gelation is limited. Moreover, it takes time (in this case, overnight) to obtain a mixture where CaCO_3_ particles are well dispersed.

**Fig. 14:**
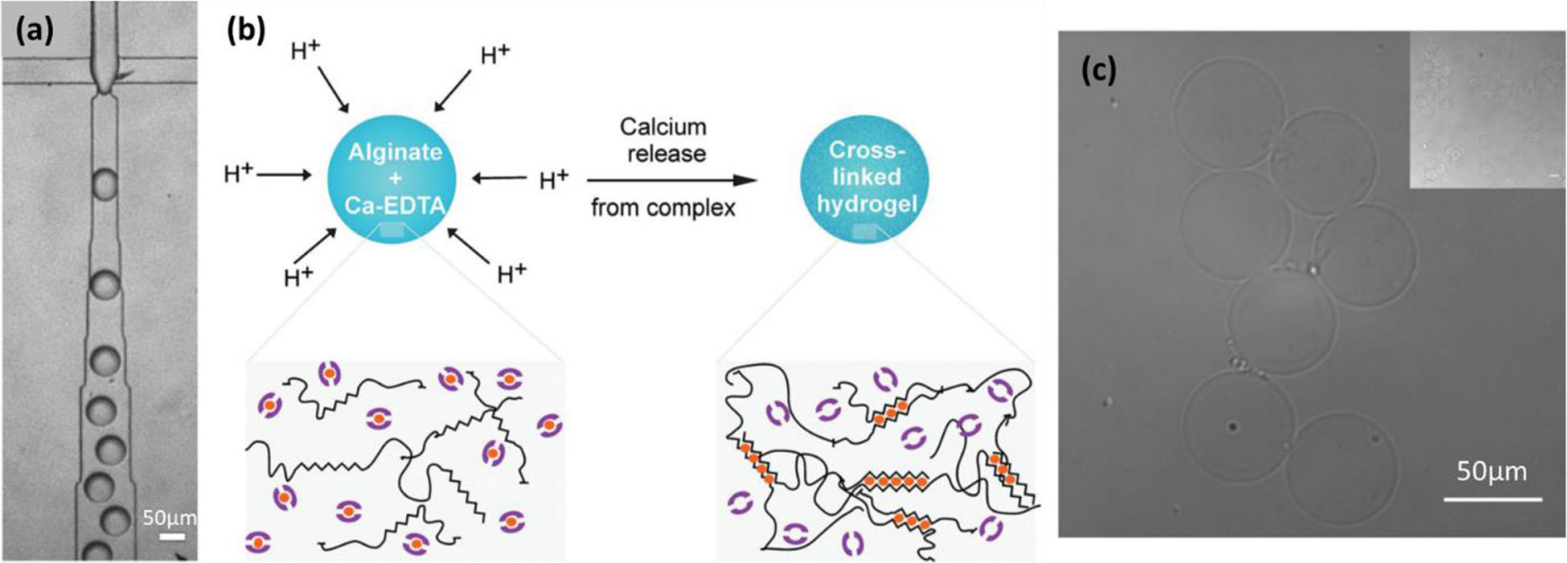
**a** Micrograph of the T-junction in a microfluidic device, where droplets of Na-alginate/Ca-EDTA were generated in oil/acetic acid. **b** Schematic illustration of the crosslinking process in each droplet. **c** Micrograph of Ca-alginate hydrogel microparticles in an aqueous medium. Figure reprinted with permission from Reference [49]

In order to obtain a homogeneous internal structure of hydrogel microparticles, Utech et al. used a slightly water-soluble calcium-ethylenediaminetetraacetic acid (Ca-EDTA) complex as the cross-linker [49]. An aqueous solution of Na-alginate (2 wt%) mixed with Ca-EDTA (50 mM) was first prepared. This homogeneous mixture was used as the dispersed fluid for the microfluidic system. The continuous fluid was a fluorinated carbon oil with a biocompatible surfactant (1 wt%) containing acetic acid (0.05 vol%). Droplets of Na-alginate/Ca-EDTA were generated in oil/acetic acid by flow-focusing (Fig. 14a). Due to the use of acetic acid, calcium cations were released from Ca-EDTA in each droplet (Fig. 14b), causing internal gelation of the alginate. The Ca-alginate hydrogel microparticles formed (Fig. 14c) had a homogeneous internal structure and were stable in an aqueous medium without dissolution. It should be noted that, the solubility of Ca-EDTA in water being low (0.26 M at 20 °C), the concentration of Ca-EDTA in the Na-alginate solution was limited in order to keep the solution homogeneous. Thus, this strategy is not appropriate when microparticles need to be highly crosslinked. Furthermore, care should be taken with Ca-EDTA, as EDTA is used to dissolve alginate hydrogel microparticles in the literature [29, 56].

In conclusion, internal gelation of alginate can be realized by using cross-linkers that are soluble or insoluble/slightly soluble in water. When water-soluble cross-linkers are used, the instant gelation disturbs droplet generation. The problem can be solved by using partially miscible fluids with limited mixing prior to droplet generation, and/or by using extremely diluted solutions and surfactant (Table 1). Mixing cross-linkers and Na-alginate after droplet generation involves merging droplets or flows of Na-alginate and water-soluble cross-linkers (Table 2). The resulting droplets are dependent on physicochemical properties like viscosity and interfacial energy. If water-insoluble/slightly soluble cross-linkers are used, they are mixed with alginate before droplet generation. For pH-sensitive cross-linkers, acid is then needed to release cations, after which internal gelation takes place (Table 3).

A homogeneous microparticle internal structure can be achieved by choosing appropriate cross-linkers. However, because of low solubility in water, it is important to limit the concentration of cross-linkers to avoid precipitates in the channel.

### External gelation

In external gelation, cross-linkers come from outside the alginate droplets and are diffused into the alginate droplets or the microparticles formed, inducing crosslinking. Unlike internal gelation, in which cross-linkers are always introduced “on-chip” (in the microfluidic device), in external gelation, cross-linkers can be introduced both “on-chip” and/or “off-chip” (outside the microfluidic device).

#### On-chip introduction of cross-linkers

For external gelation, several authors introduced cross-linkers “on-chip”. They used calcium acetate (Ca (CH_3_COO)_2_) or CaCl_2_ as cross-linkers, as summarized in Table 4.

**Table 4:** External gelation with on-chip introduction of cross-linkers

Reference		Zhang et al., 2007 [61]	Liu et al., 2019 [29]	Sugaya et al., 2011 [44]	Pittermannová et al., 2016 [34]
**Droplet generation**	**Concentration of Na-alginate**	2 wt%	3 wt%	0.025–0.15 wt%	1 w/v%
	**Continuous fluid**	Soybean oil	Corn oil	Methyl acetate	1-undecanol
	**Use of surfactant**	Span 80	SY-Glyster CRS-75	Not mentioned	Abil Em 90
	**Geometry**	Flow-focusing	Flow-focusing	Flow-focusing	Flow-focusing
	**Microfluidic material**	PDMS chip	Glass chip	PDMS chip	PDMS chip
**External gelation by introducing Na-alginate and cross-linkers on chip before collection**	**Cross-linkers**	Ca (CH_3_COO)_2_ (2 wt%) in the continuous fluid	Emulsion of CaCl_2_ in the continuous fluid	CaCl_2_ (1 M) in the dispersed fluid	Emulsion of CaCl_2_ (2 wt%) droplets in the continuous fluid
	**Geometry**	–	Flow-focusing	Cross-flowing	Flow-focusing
	**Mixing**	During droplet generation	After droplet generation	After droplet generation	After droplet generation

**Fig. 15:**
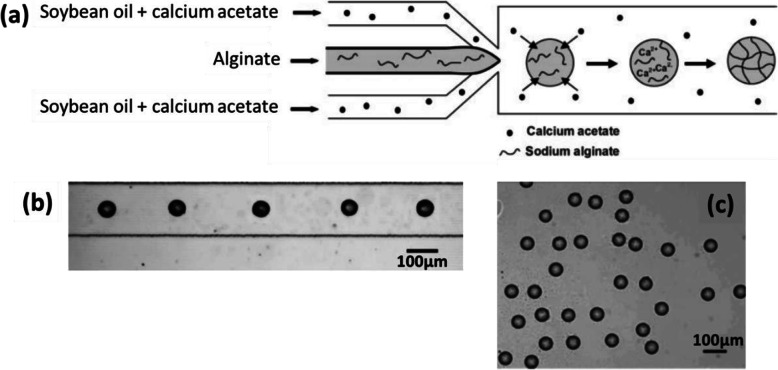
**a** Schematic diagram of the preparation of Ca-alginate hydrogel microparticles via on-chip external gelation in a PDMS-based microfluidic device. Micrographs of Ca-alginate hydrogel microparticles **b** in the downstream channel and **c** in the collecting container in soybean oil. Figure reprinted with permission from Reference [61]

Cross-linkers can be contained in the continuous fluid, as described in Zhang et al. [61] Ca (CH_3_COO)_2_ (2 wt%) was dissolved in soybean oil, the continuous fluid. In the microfluidic device detailed previously [61], Na-alginate (2 wt%) droplets were generated by flow-focusing (Fig. 15a) in oil/Ca (CH_3_COO)_2_, with surfactant (Span 80, 3 wt%). Ca (CH_3_COO)_2_ diffused and dissolved in Na-alginate droplets along the channel (Fig. 15b), causing external gelation on-chip. Finally, Ca-alginate hydrogel microparticles were collected in oil (Fig. 15c). They showed better stability in an aqueous medium and had a higher Young’s modulus compared with those produced by internal gelation (III.1.2.). Consequently, stronger gelation was achieved by external gelation. However, increasing the concentration of Ca (CH_3_COO)_2_ in soybean oil caused clogging in the microchannel [61]. Thus, it is difficult to vary the rate of gelation of microparticles.

**Fig. 16:**
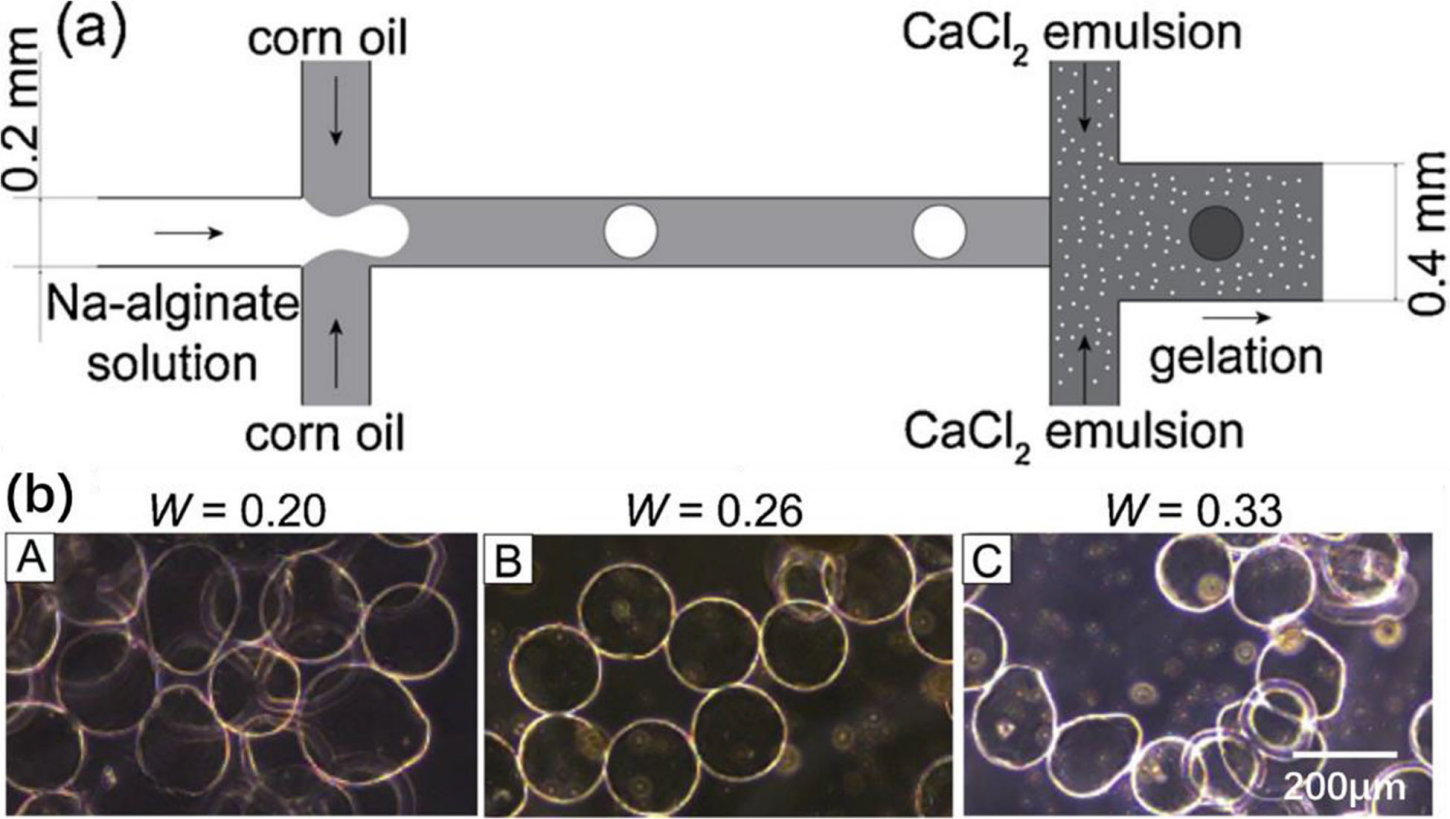
**a** Schematic diagram of a glass-based microfluidic device for the preparation of Ca-alginate hydrogel microparticles, with channels modified so as to be hydrophobic. **b** Micrographs of hydrogel microparticles obtained with different mass fractions of the aqueous CaCl_2_ solution in emulsion (W). Figure reprinted with permission from Reference [29]

One way to limit channel clogging is to make the cross-linkers diffuse slowly in Na-alginate droplets. Thus, Liu et al. used emulsion fluids to introduce cross-linker s[29]. A glass-based microfluidic device was used, with channels modified so as to be hydrophobic. Droplets of Na-alginate (3 wt%) were first generated in corn oil at the first flow-focusing channel (Fig. 16a). The emulsion of CaCl_2_, containing CaCl_2_ droplets in corn oil (with surfactant SY-Glyster CRS-75), was injected downstream of the cross-junction. The contact between CaCl_2_ and Na-alginate droplets caused ionic crosslinking, leading to gelation. Ca-alginate hydrogel microparticles were obtained. However, it was found that the microparticles could easily be deformed (Fig. 16b-A, b-C) by several parameters, such as the mass fraction of the aqueous CaCl_2_ solution in emulsion (W). Deformation occurred when the value of W was too high or too low, so that an optimal value of W was required for homogeneous spherical microparticles (Fig. 16b-B). The morphology and homogeneity of microparticles also varied with flow rates and surfactant concentrations. Lacking flexibility, this strategy is thus not appropriate for producing spherical hydrogel microparticles. Moreover, generating small particles requires reducing the channel size, involving a risk of droplet coalescence before gelation in CaCl_2_ emulsion.

To avoid reducing the channel size, partially miscible fluids can be used as the dispersed and continuous fluids. Sugaya et al. used methyl acetate as the continuous fluid [44]. Na-alginate (0.025–0.15 wt%) droplets were generated in methyl acetate (no mention of surfactant usage) by flow-focusing. In the following channel, because of the solubility of water in methyl acetate (8 wt%), water dissolved gradually from the droplets into methyl acetate. Thus, the droplets shrank and became more concentrated downstream. CaCl_2_ solution (1 M) was then injected by side channels and flowed with the droplets by co-flow. Calcium cations diffused into the droplets, inducing on-chip external gelation of alginate. Finally, spherical Ca-alginate hydrogel microparticles with a diameter of less than 20 μm were obtained. In this strategy, after CaCl_2_ fluids were introduced, two competing processes occurred simultaneously in each droplet: gelation and shrinkage of droplets. The competition between gelation and shrinkage is not discussed in this article. However, the results indicate that extremely small droplets tend to approach the channel wall after shrinkage. With the CaCl_2_ fluid, after gelation, Ca-alginate hydrogel microparticles adhere to the channel wall.

**Fig. 17:**
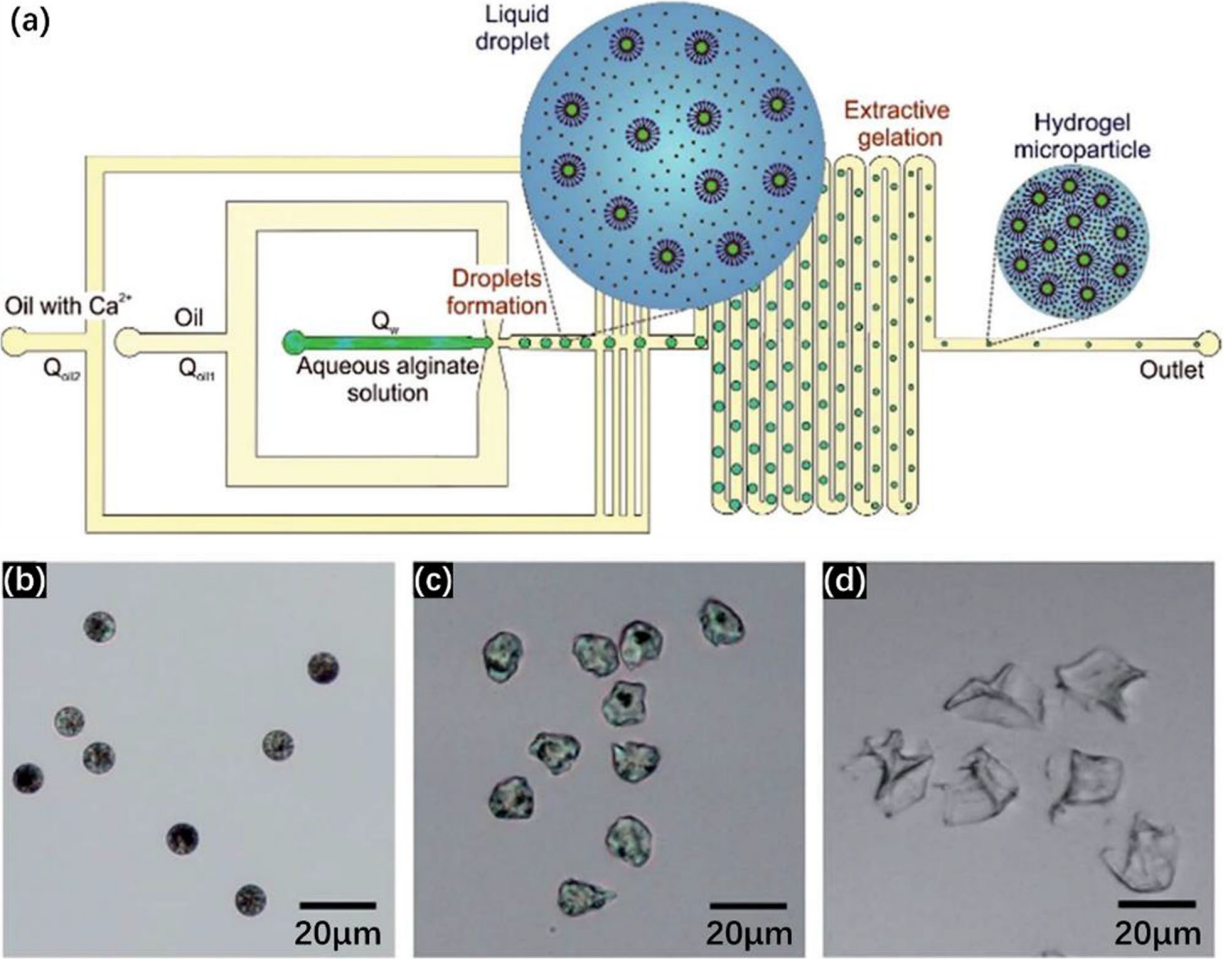
**a** Schematic diagram of a PDMS-based microfluidic device for the preparation of Ca-alginate hydrogel microparticles. Micrographs of microparticles with a **b** spherical, **c** slightly deformed and **d** collapsed morphology obtained using different experimental flow rates and calcium concentrations. Figure reprinted with permission from Reference [34]

Adhesion to the channel and coalescence of microparticles can be avoided thanks to progressive addition of the cross-linker. Pittermannová et al. used as continuous fluid 1-undecanol, whose water-solubility is 2.7 vol% [34]. The experiment was carried out in a PDMS-based microfluidic device (Fig. 17a). An aqueous alginate solution (1 wt%) was first injected. After 1-undecanol (with 5 wt% surfactant Abil Em 90), shown as “oil” in Fig. 17a, was injected into the flow-focusing channel, droplets of alginate were formed. CaCI_2_ (2 wt%) was dispersed in another fluid, 1-undecanol with 5 wt% surfactant Abil Em 90, yielding an emulsion. This emulsion was injected after droplet generation, through successive channels (Fig. 17a). Hence, the droplets were increasingly separated from each other, avoiding coalescence. Moreover, they were surrounded by more and more CaCl_2_, increasing gelation, and by more and more 1-undecanol, increasing diffusion of water. Thus, gelation and shrinkage of droplets occurred gradually and simultaneously. According to the authors, this procedure avoids the droplet generation instability caused by pre-gelation. However, spherical hydrogel microparticles (Fig. 17b) were only obtained using certain flow rates and calcium concentrations. Otherwise, the microparticles were slightly deformed (Fig. 17c) or collapsed (Fig. 17d), which was explained using a core-shell model [34]. Unfortunately, this explanation does not take into account the change in alginate concentration in the droplets due to water extraction, a factor which is bound to impact deformation. Simply prolonging the water extraction process before introducing cross-linkers, as done by Sugaya et al., could avoid deformation [44].

#### Off-chip introduction of cross-linkers

Other strategies of external gelation introduce cross-linkers “off-chip”, i.e., during droplet collection. Ca (CH_3_COO)_2_ or CaCl_2_ are used as cross-linkers and the collection bath procedure depends on the strategy, as summarized in Table 5.

**Table 5:** External gelation with off-chip introduction of cross-linkers

References		Hu et al., 2012 [20]		Zhang et al., 2020 [59]	Present review	
**Droplet generation**	**Na-alginate**	1.5 wt%		0.006–1 wt%	0.006–1 wt%	
	**Continuous fluid**	N-decanol		DMC^a^	DMC^a^	
	**Use of surfactant**	Span 80		NO	NO	
	**Geometry**	Flow-focusing		Cross-flowing	Cross-flowing	
	**Microfluidic material**	Glass chip		Fluoropolymer tubing and junctions	Fluoropolymer tubing and junctions	
**External gelation by introducing Na-alginate and cross-linkers off-chip during collection**	**Number of phases or steps**	2 phases - 1 collection bath		1 phase - 1 collection bath	2 phases - 2 successive collection baths	
	**Description of phases or steps**	Phase 1	CaCl_2_ (15 wt%) in n-decanol	CaCl_2_ (0.1–1 wt%)	Step 1	DMC and evaporation of DMC
		Phase 2	Ba (CH_3_COO)_2_ (15 wt%) in water and glycerol		Step 2	CaCl_2_ (0.5–10 wt%) in water
	**Surfactant**	Span 80		NO	NO	

^a^Continuous and dispersed fluids partially miscible

**Fig. 18:**
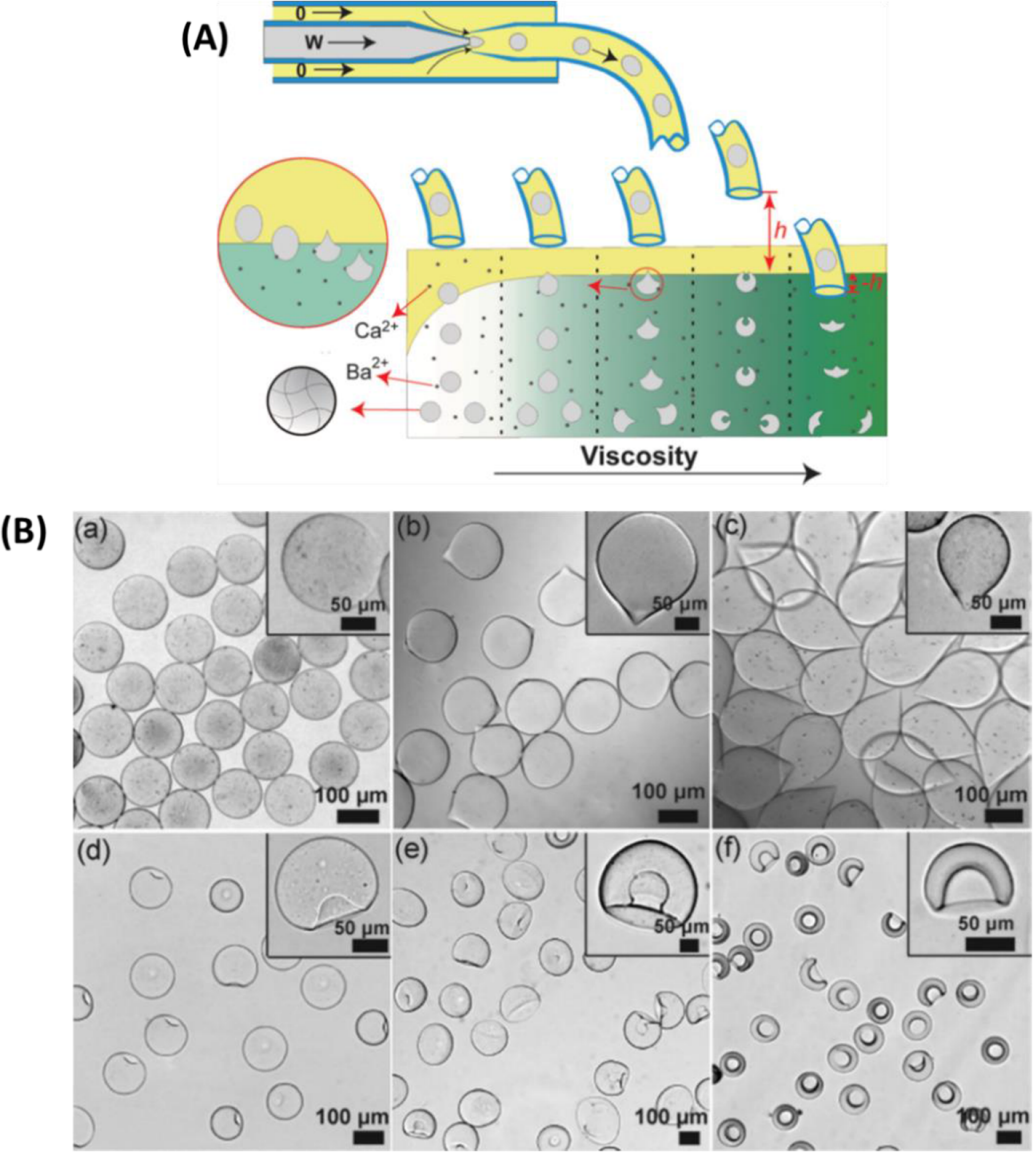
**A** Schematic diagram of the preparation of Ca-alginate hydrogel microparticles using a microfluidic device constructed with glass capillaries, and off-chip gelation in a two-phase gelation bath. **B** Micrographs of Ca-alginate hydrogel microparticles of different shapes prepared under different experimental conditions. Figure reprinted with permission from Reference [20]

Hu et al. [20] studied the influence of external gelation conditions on the shape of microparticles. Na-alginate (1.5 wt%) droplets were first generated in n-decanol with surfactant (Span 80, 5 wt%), using concentric glass capillaries in co-flow geometry (Fig. 18A). For off-chip external gelation, droplets were collected in a two-phase gelation bath: the upper phase of n-decanol with surfactant (Span 80, 5 wt%) containing CaCl_2_ (15 wt%) allowed for pre-gelation of alginate; the bottom phase, an aqueous solution of barium acetate (15 wt%), strengthened the gelation. Glycerol (0–70 wt%) was added to the bottom phase to regulate viscosity. Ca-alginate hydrogel particles of different shapes (Fig. 18B) were obtained by varying gelation conditions such as the interfacial energy γ_CD_, the concentration and type of surfactant, the height h between the end of the capillary and the surface of the gelation bath, and the viscosity of the bottom phase in the gelation bath. The shape of microparticles was shown to depend on forces applied to the surface of droplets when they passed through the interface in the gelation bath. The force from γ_CD_ maintains the spherical form of droplets, while the viscous force causes deformation. The final shape resulted from the overall effect of these two forces [20]. As can be seen in this strategy, droplet collection is accompanied by the consumption of the two different cations. Thus, to obtain a large quantity of microparticles, these cations should be replenished to ensure that each droplet undergoes sufficient gelation. However, in practice, when and how to replenish them remains an issue.

**Fig. 19:**
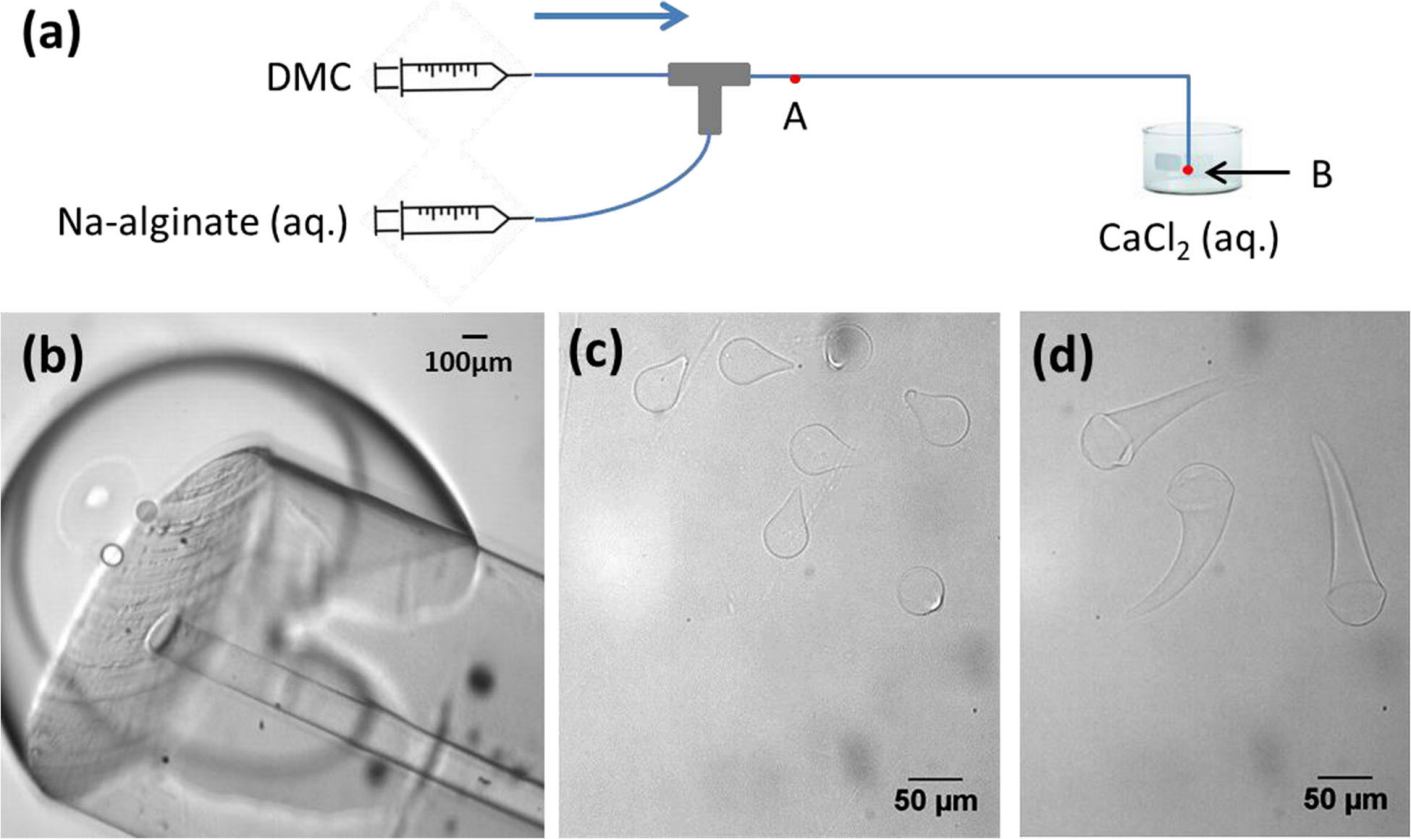
**a** Schematic diagram of the preparation of Ca-alginate hydrogel microparticles by off-chip external gelation without pre-gelation. Droplets were generated using a microfluidic device assembled from fluoropolymer capillaries and a T-junction. Micrographs of **b** the channel outlet immersed in an aqueous solution of CaCl_2_; Ca-alginate hydrogel microparticles prepared by collecting droplets in an aqueous solution of CaCl_2_ at concentrations of **c** 1 wt% and **d** 0.1 wt%

To avoid the problem of replenishing the bath with the two cations, we collected Na-alginate droplets in an aqueous solution containing CaCl_2_ (Fig. 19a) without pre-gelation. Na-alginate (0.006–1 wt%) droplets were first generated in DMC in a T-junction and Teflon-like capillaries (IDEX Health and Science), without using surfactant [59]. Because water is slightly soluble in DMC, 3 wt%, water diffused gradually from droplets into DMC, causing the droplets to shrink as they passed through the channel (point A to B in Fig. 19a). Thus, droplet size reduced to below 100 μm. Furthermore, since alginate dissolution in the continuous fluid is negligible [39], with the loss of water, the alginate concentration in droplets increased. Then, the channel outlet (point B in Fig. 19a) was immersed in an aqueous solution of CaCl_2_ (0.1–1 wt%). An interface was created at the channel outlet (Fig. 19b) because of the non-total miscibility between DMC and water. Na-alginate droplets passed through the interface and entered the CaCl_2_ solution, leading to off-chip external gelation. After gelation, Ca-alginate hydrogel microparticles were droplet-shaped (Fig. 19c) and tadpole-shaped (Fig. 19d), as in Fig. 18B, b-c. The shape of the microparticles varied with the flow rates, the concentration of Na-alginate and that of CaCl_2_. It is likely that the deformation mechanism involved the forces applied to droplets at the interface, as explained by Hu et al. [20].

**Fig. 20:**
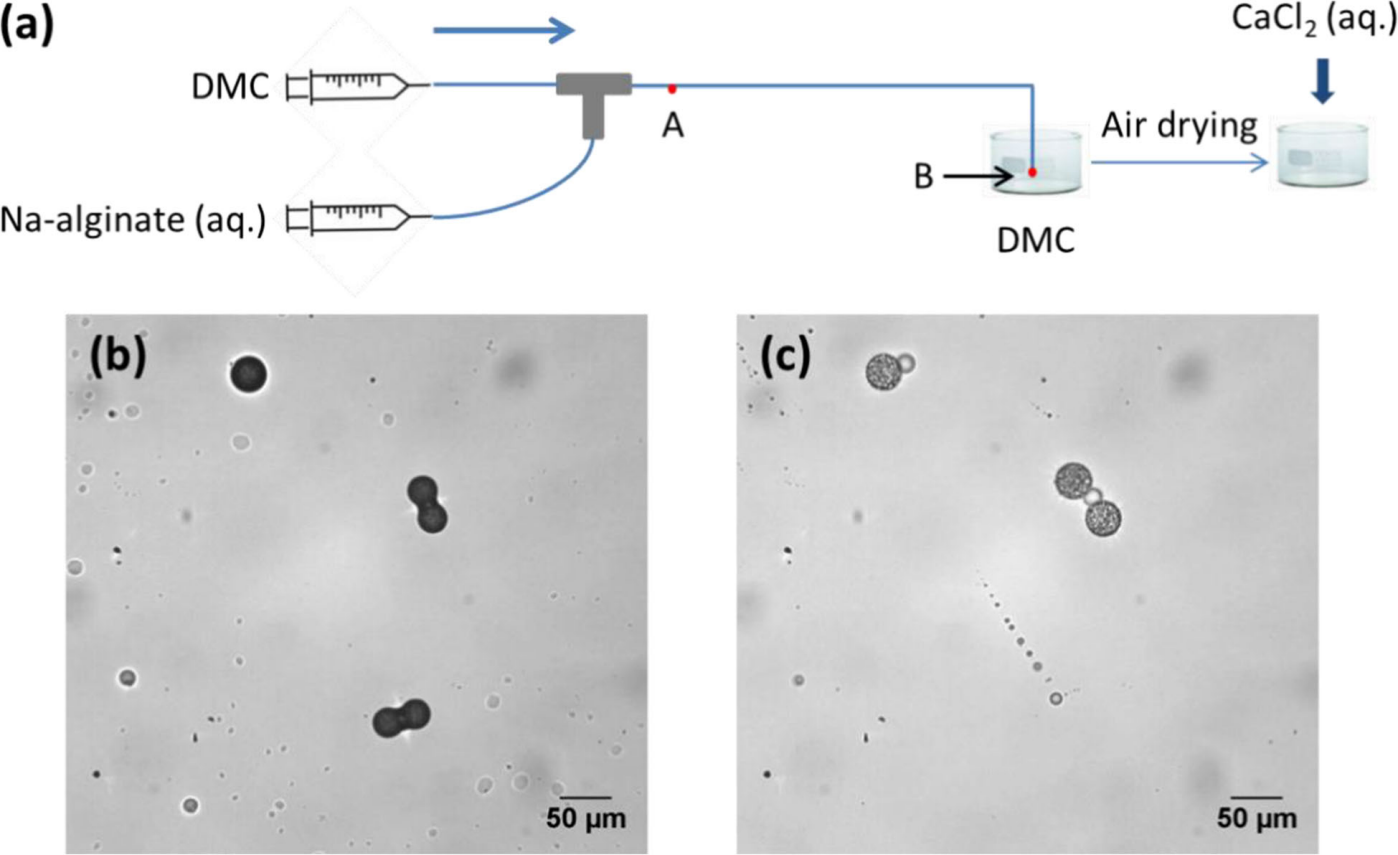
**a** Schematic diagram of a two-step preparation of Ca-alginate hydrogel microparticles using a microfluidic device constructed from fluoropolymer capillaries and junctions. Micrographs of **b** dried Na-alginate microparticles in air and **c** corresponding Ca-alginate microparticles after gelation in CaCl_2_ solution. Figure reprinted and adapted with permission from Reference [59]

To improve the spherical shape of microparticles, we collected droplets in a bath of the continuous fluid, i.e., DMC. Hence, the droplets continued to shrink and were finally transformed into spherical condensed Na-alginate microparticles, not yet gelated. For the gelation of the microparticles, the bath of DMC was first evaporated. Then, an aqueous solution of CaCl_2_ (0.5–10 wt%) was added to the dried Na-alginate microparticles, inducing off-chip external gelation. Observations showed that this process was accompanied by the swelling of the microparticles without deformation (Fig. 20b-c). In the end, spherical Ca-alginate hydrogel microparticles were obtained. They were insoluble in water, indicating efficient gelation. Moreover, the concentration of CaCl_2_ had no significant effect on the size of the Ca-alginate microparticles. Since no surfactant is used in this method, no surfactant-removing step is needed, which simplifies the process. However, the quantity of microparticles produced is limited by the need to avoid droplet coalescence.

In conclusion, external gelation of alginate can be performed both on-chip and off-chip. For on-chip external gelation (Table 4), cross-linkers can be added to the continuous fluid, i.e., the oil. However, only limited concentrations can be used, since most are slightly soluble in oil. Therefore, introducing cross-linkers in emulsion form increases the quantity of alginate available for gelation. However, the particles are large and deformed. On the other hand, if partially miscible fluids are used, an aqueous solution of cross-linkers can be injected after droplet shrinkage. Particle size is reduced but the gelation is too rapid. Things can be improved by dissolving cross-linkers in an oil-based emulsion, introduced in small quantities but repeatedly.

For off-chip external gelation (Table 5), cross-linkers are introduced into the collection bath. A two-phase collection bath permits pre-gelation of Na-alginate droplets before gelation. However, the particles are large. Droplet size can be reduced to below the channel diameter by using partially miscible fluids for droplet generation, and the droplets can then be collected directly in the dispersed phase containing the cross-linker. However, the microparticles are deformed. To further reduce particle size and improve gelation, our solution is to perform two-step collection. Thus, off-chip external gelation can be used to produce shape-controlled and size-controlled microparticles.

## Properties of alginate hydrogel microparticles

After preparation, alginate hydrogel microparticles should be characterized to obtain better knowledge of their properties, which will determine their further applications. This section discusses characterization approaches and factors influencing particle properties.

### Size

Size is one of the most important properties of alginate hydrogel microparticles. For example, in drug delivery, microparticle size and size distribution affect drug release kinetics [50]. Size can be measured by optical or light-scattering (sub-micrometer range) microscopy [21], or using microgrippers [58].

Droplet-based microfluidics allows monodisperse microparticles to be produced with accurate control of size and size distribution. Table 6 shows the average size attained under droplet-based microfluidics using different gelation methods.

**Table 6:** Average size of alginate microparticles prepared using droplet-based microfluidics with different gelation methods

Average size of microparticles	Gelation method	Reference
1–50 μm 10–300 nm	Internal gelation with water-soluble cross-linkers mixed with Na-alginate before droplet generation	Rondeau and Cooper-White 2008^a^ [39]
50–300 μm	Internal gelation with water-soluble cross-linkers mixed with Na-alginate after droplet generation	Xu et al. 2008 [54]
20–50 μm		Liu et al. 2006 [28]
22–42 μm		Trivedi et al. 2009 [48]
60–100 μm	Internal gelation with water-insoluble cross-linkers mixed with Na-alginate after droplet generation	Zhang et al. 2007 [61]
26 μm		Akbari and Pirbodaghi 2014 [3]
50–100 μm		Yu et al. 2019 [56]
10–50 μm	Internal gelation with slightly water-soluble cross-linkers mixed with Na-alginate after droplet generation	Utech et al. 2015 [49]
50–70 μm	External gelation with on-chip introduction of cross-linkers	Zhang et al. 2007 [61]
147–176 μm		Liu et al. 2019 [29]
5–10 μm		Pittermannová et al. 2016^a^ [34]
6–10 μm		Sugaya et al. 2011^a^ [44]
100–200 μm	External gelation with off-chip introduction of cross-linkers	Hu et al. 2012 [20]
7–40 μm		Zhang et al. 2020^a^ [59]

^a^Partially miscible fluids were used

**Fig. 21:**
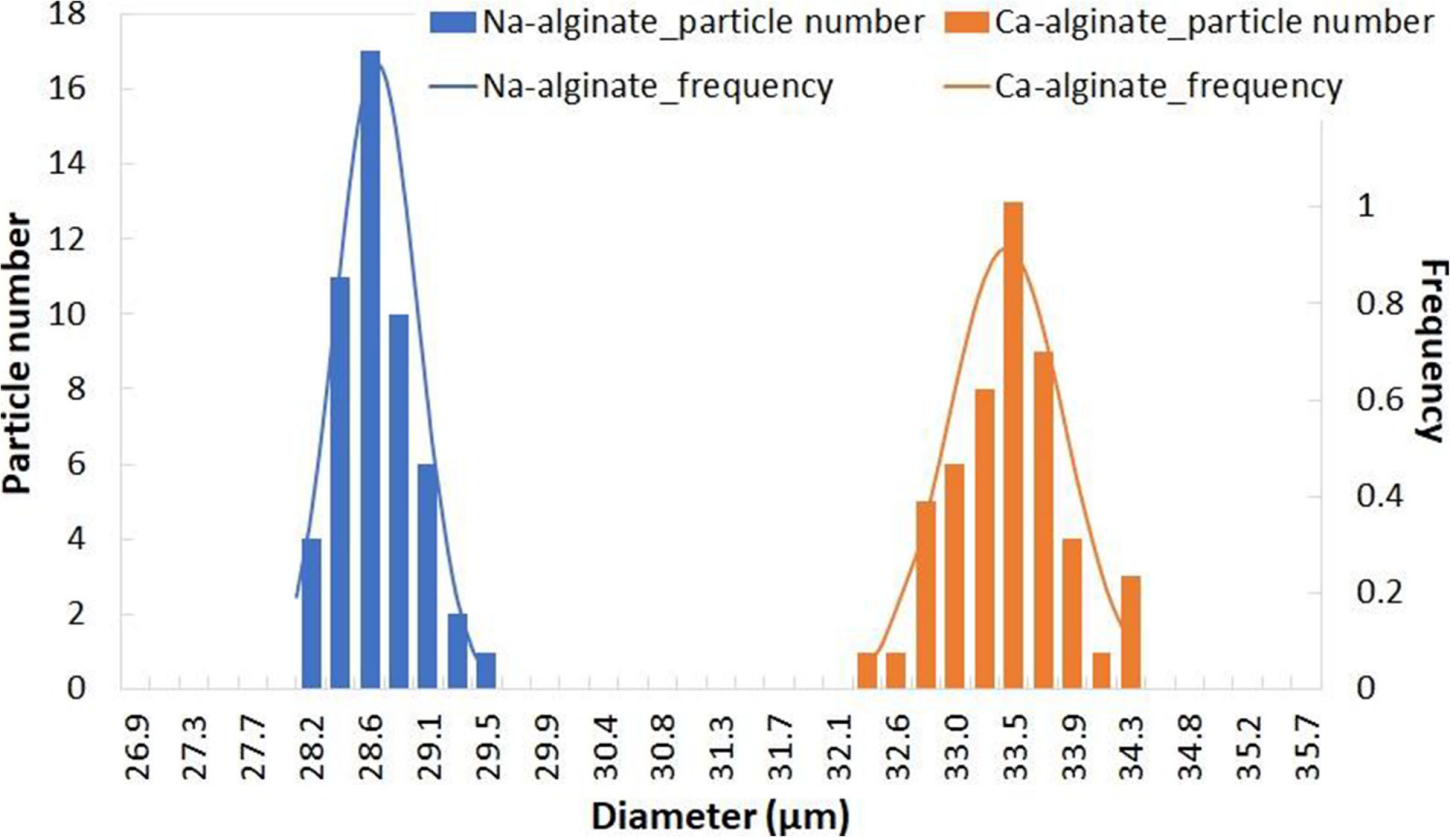
Size distribution of microparticles of Na-alginate (blue) and Ca-alginate (orange) produced using droplet-based microfluidics. The curves show Gaussian fitting. Figure reprinted and adapted with permission from Reference [59]

Figure 21 shows an example of the narrow size distribution of Na-alginate and Ca-alginate microparticles produced by droplet-based microfluidics [59] (part III.2.2.), indicating the monodispersity of the particle size. This is an advantage compared to conventional emulsification, which yields a broader size distribution [55].

With droplet-based microfluidics, the size of alginate hydrogel microparticles is influenced by several factors linked to the fluids used to generate them. When immiscible fluids are used for the dispersed and the continuous fluids, the size of alginate hydrogel microparticles is completely dependent on the size of the droplets first generated. Droplet size is influenced by channel size, and smaller droplets can be generated by using narrower channels. Other important factors are flow rates, alginate concentration [39, 59], and fluid viscosities [41, 46]. However, reducing channel size increases hydraulic resistance, as well as the pressure required to generate droplets. Moreover, it should be noted that in most cases, the Na-alginate solution used is relatively viscous. Therefore, high pressure may cause leakage or even destruction of the microfluidic device [3, 49]. Thus, even when channel diameter is decreased and/or the flow rate of the continuous fluid is increased, producing droplets of a diameter below 10 μm remains challenging.

Droplets of this size, below 10 μm, can be obtained without applying high pressure (Table 6), by using partially miscible fluids with low solubility in each other [39, 44, 59]. The dispersed fluid is an aqueous solution containing Na-alginate; the continuous fluid is an organic solvent that is partially miscible with water and in which water has low solubility. The partial miscibility between the continuous and the dispersed fluids should be slight enough so that interfacial energy γ_CD_ still allows the generation of droplets. The low solubility of water in the continuous fluid allows water diffusion from droplets into it, causing the droplets to shrink. As a result, the initially obtained diluted large droplets are transformed into concentrated small droplets or microparticles. Thus, their size is no longer dependent on the size of droplets initially generated but varies with the interaction between water and the continuous fluid.

### Shape

The shape of microparticles is another important property. A specific shape is sometimes needed; for example, red blood cell-mimicking microparticles are often required in a biconcave shape [32]. In drug delivery, the shape of microparticles has an impact on the drug-release profile [13]. The overall shape of microparticles can be observed by using optical microscopy. Confocal microscopy of fluorescent samples can be used to form a spatial 3D image [21]. Better resolution can be obtained by using atomic force microscopy (AFM) or scanning electron microscopy (SEM) [59].

With droplet-based microfluidics, the spherical droplets initially generated can be transformed into spherical alginate hydrogel microparticles after gelation. Non-spherical microparticles can also be obtained. For example, as presented previously, Liu et al. first generated droplets of Na-alginate and CaCl_2_ separately [28]. Then the droplets were fused in a specifically designed microfluidic device, leading to gelation. By varying the channel geometry and controlling the flow rates of fluids, Ca-alginate microparticles of different shapes were obtained (Fig. 10f). A different method was presented by Hu et al. [20] Na-alginate droplets were first generated and then collected in a two-phase gelation bath. Spherical droplets were deformed via interfacial energy derived from surfactant and viscous force. Thus, different shapes were produced (Fig. 18B) by controlling the surfactant used and the viscosity of the gelation bath.

### Concentration

After preparation, the concentration of alginate in the microparticles can be calculated approximately. For instance, Zhang et al. used partially miscible fluids [59]. An aqueous solution of Na-alginate was prepared with a known concentration. After droplet generation, droplet shrinkage occurred during passage through the channel due to water diffusion into the continuous fluid. Droplets were hence transformed into microparticles. As the diffusion of Na-alginate into the continuous fluid is negligible [39], the quantity of Na-alginate is constant. It can be calculated by multiplying the droplet volume and initial concentration. Finally, by measuring microparticle size, the concentration of Na-alginate can be calculated. The final concentration of Na-alginate varies from 20 to 100 wt%, depending on the initial concentration and diameter of the droplets generated [59]. Furthermore, as presented in Utech et al., the homogeneity of composition of microparticles can be determined with the help of fluorescence technology [49].

### Stability

In most cases, surfactant is added in the continuous fluid [48, 49, 61] to lower interfacial energy between the continuous and the dispersed fluids γ_**CD**_. Note that for each of the above studies, use or non-use of surfactant is mentioned when indicated by the authors (Tables 1, 2, 3, 4, 5). Surfactant facilitates the creation of a new interface, and thus the formation of droplets. It also stabilizes the formed droplets by preventing their coalescence [41]. Before the application of microparticles, the surfactant should be dissolved [3], except for biocompatible surfactant [49], although protocols for removing surfactant are rarely reported in the literature. To remove the oil used during the preparation, microparticles should be washed several times with an aqueous solution, followed by centrifugation [61].

However, despite its advantages, the use of surfactant may be undesirable. Surfactant has been shown to impact the surface properties of microparticles, such as morphology [45] and surface hydrophobicity [22]. Additionally, if rinsing is insufficient, the traces of surfactant on microparticles can damage the devices during application. In this situation, microparticles should be prepared without surfactant, which is possible with droplet-based microfluidics. In the microchannel, the coalescence of droplets can be avoided by enlarging the distance between droplets, which can be achieved by regulating flow rates. Furthermore, gelation, either on-chip or off-chip, solidifies droplets and thus helps to avoid coalescence as well. Another strategy consists of using partially miscible fluids. This means that the droplets shrink and become more and more condensed during passage through the channel. At the outlet, either gelation [39] or a final shrinkage [59] can help avoid coalescence.

Moreover, for their stability, alginate hydrogel microparticles should be insoluble in water. This can be achieved by adopting proper gelation methods using a sufficient quantity of cross-linkers for effective gelation.

### Mechanical properties

Mechanical properties of alginate hydrogel microparticles are usually characterized by measuring the Young’s modulus, which varies with several factors. According to the type of bond between alginate and cross-linkers, covalent crosslinking results in a higher Young’s modulus than ionic crosslinking in microparticles [6]. For ionic crosslinking, different cations present different affinities, i.e. different forces with alginate, thus different Young’s moduli [33]. In addition, the Young’s modulus increases with the concentration of alginate [31]. To measure the Young’s modulus of a microparticle, it needs to be deformed under a known force, which can be either compressive or tensile [17]. The techniques used in the literature include micropipette aspiration [23], compression [7, 52] or Atomic Force Microscopy (AFM) [59, 61].

#### Micropipette aspiration technique

In the micropipette aspiration technique, controlled pressure is used to pull on the sample surface. When this pressure is high enough, the sample behaves like a viscoelastic fluid flowing inside the micropipette [17]. With a known pressure applied, the Young’s modulus is calculated by applying the homogenous half-space model as described by Kleinberger et al. [23].

#### Compression technique

This technique consists in compressing a microparticle between two parallel plates [7] or between the flat end of a glass fiber and a glass surface [52]. A force transducer is connected to the equipment to measure the force applied. By varying the force, microparticle deformation can be recorded. Finally, according to the force-deformation curve and equations based on theoretical models, the Young’s modulus is calculated.

However, both the micropipette aspiration technique and the compression technique are unsuitable for microparticles with high resistance to deformation, like the Na- and Ca-alginate microparticles generated by Zhang et al. [59]. In this case, Atomic Force Microscopy was used to measure resistance to deformation.

#### Atomic force microscopy

**Fig. 22:**
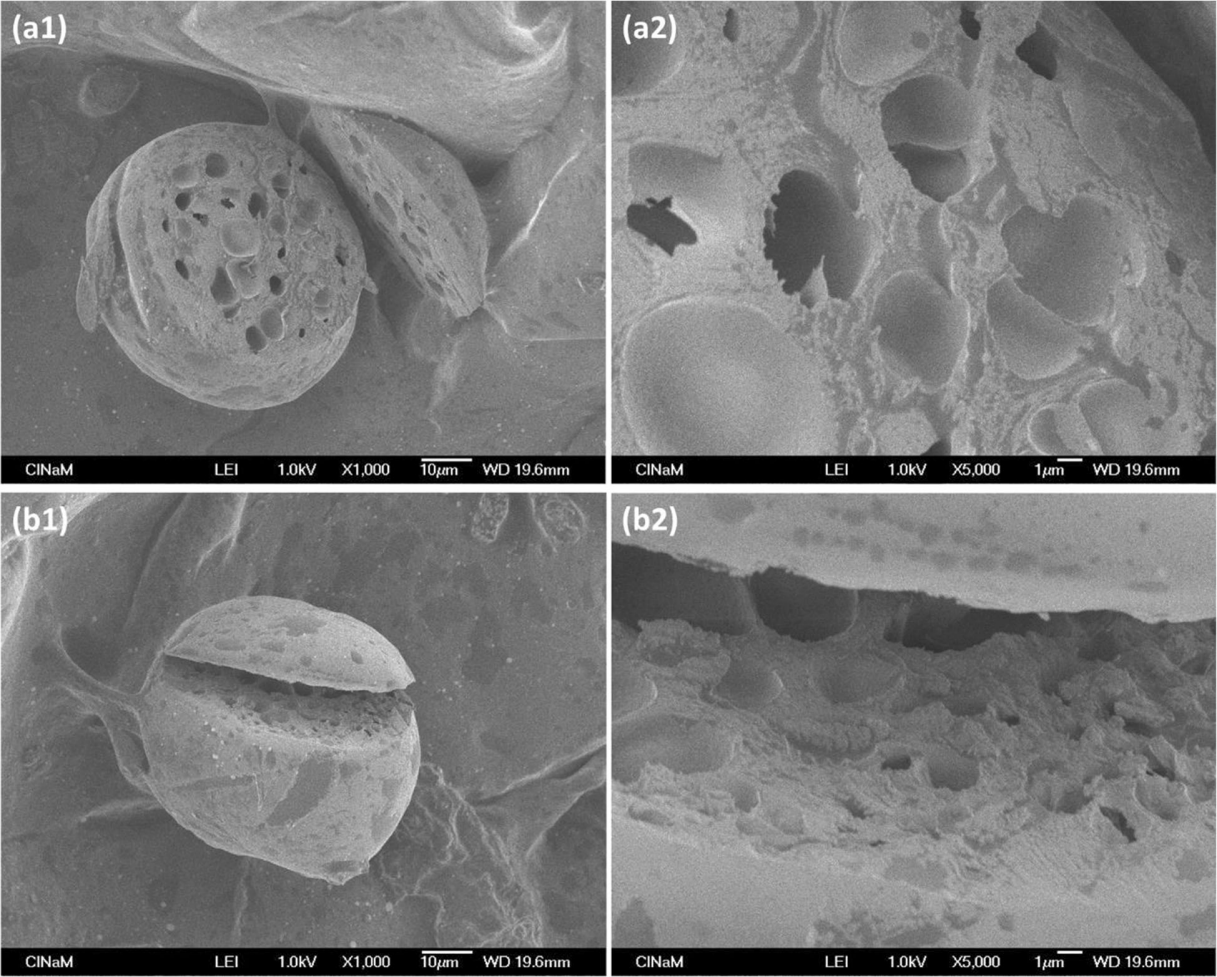
SEM photographs of 2 Na-alginate microparticles (**a**) and (**b**), magnified 1000x (**a1** and **b1**) and 5000x (**a2** and **b2**). Na-alginate microparticles were prepared following the method mentioned in the publication [59]. Figure reprinted and adapted with permission from Reference [59]

Measurement of the Young’s modulus of a microparticle with Atomic Force Microscopy (AFM) involves indenting it. The indentation depth (order of 100 nm) is generally about 100 times less than the diameter of the microparticle (order of 10 μm). Hence the Young’s modulus represents the local mechanical property on the surface, depending on the measuring point, as in Zhang et al. [59]. As the surface of their microparticles was smooth, variation in the local mechanical property was explained by the porous inner structure observed by Scanning Electron Microscopy (SEM) (Fig. 22).

## Conclusion

This review focuses on the preparation of alginate hydrogel microparticles via droplet-based microfluidics. Various strategies are presented and classified within categories that represent the full range of methods used in the literature. Thus, readers will find that any strategy encountered can fit into one of the categories we present here.

To summarize, gelation is indispensable to transform alginate microdroplets into alginate hydrogel microparticles. It is realized by crosslinking, which requires cross-linkers to be introduced either inside or outside the microdroplets, causing respectively internal gelation or external gelation. For internal gelation, cross-linkers are introduced “on-chip” (in the microfluidic device). For external gelation, cross-linkers can be introduced both “on-chip” and/or “off-chip” (outside the microfluidic device). The review describes the various strategies applied under the microfluidic technique, and the size, shape, concentration, stability and mechanical properties of the alginate hydrogel microparticles obtained.

Lastly, we wish to stress the ease of constructing a microfluidic device with Fluoropolymer tubing and junctions compared to a chip, whatever its composition: PDMS, PMMA or glass. A microfluidic device’s geometry can be tuned flexibly, whereas in a chip, the geometry of channels is fixed. Chip fabrication is laborious, time-consuming and expensive [12]. Clean room facilities are indispensable and the products used for the photolithography are toxic.

Thus, in terms of flexibility, cost and efficiency, we highly recommend using a microfluidic device with Fluoropolymer tubing and junctions, especially for proof-of-concept demonstrations.

## Outlook

There is scope for several future improvements in the preparation of alginate hydrogel microparticles via droplet-based microfluidics.

1. When preparing microparticles using droplet-based microfluidics, surfactant is usually used. Unfortunately, surfactant has been shown to affect particle morphology [34]. Moreover, surfactant can damage the equipment involved in subsequent applications [59]. It obviously needs to be removed; however, the literature contains little information on how to remove surfactant. Moreover, despite its importance, the microparticle purifying process is barely touched on [34]. Future work could therefore usefully provide more details of the full preparation process, including surfactant removal.
2. In practice, a large number of microparticles usually needs to be produced. However, the throughput of microparticles fabricated by microfluidics is still limited. Exploring ways to scale up microparticle production would therefore be a welcome contribution.
3. Currently, most publications concern proof-of-concepts or preliminary demonstrations. Little work is available on microparticles in real biological or biomedical applications [53]. Apart from biological barriers [35], the physiological environment is quite complex in terms of composition and rheological characteristics, which makes it extremely unlikely that microparticles can provide the functions required. The feasibility of using alginate hydrogel microparticles in real-life applications needs to be assessed.
4. The relation between degree of gelation and mechanical properties is rarely discussed in the literature. In fact, the degree of gelation itself is barely mentioned although, from a microscopic point of view, it influences mechanical properties. Thus, we believe it is worth assessing degree of gelation by measuring the concentration of cross-linkers inside the final hydrogel microparticles. Subsequently, the relation between degree of gelation and mechanical properties should also be addressed.
5. In practical applications, the surface of microparticles usually needs to be modified. For example, one way to detect antigens is to graft antibodies onto the surface of microparticles [38]. We note that the surface modification is always an extra step after the preparation of microparticles, thus complicating the process. The feasibility of an “all-in-one” process should be studied: can all the steps be performed within one single microfluidic device, combining microparticle production and surface modification?

## Data Availability

Not applicable.
